# *Mycobacterium tuberculosis* Nucleoside Diphosphate Kinase Inactivates Small GTPases Leading to Evasion of Innate Immunity

**DOI:** 10.1371/journal.ppat.1003499

**Published:** 2013-07-18

**Authors:** Jim Sun, Vijender Singh, Alice Lau, Richard W. Stokes, Andrés Obregón-Henao, Ian M. Orme, Dennis Wong, Yossef Av-Gay, Zakaria Hmama

**Affiliations:** 1 Division of Infectious Diseases, Department of Medicine and Vancouver Coastal Health Research Institute, University of British Columbia, Vancouver, British Columbia, Canada; 2 Life Sciences Centre, Department of Microbiology and Immunology, University of British Columbia, Vancouver, British Columbia, Canada; 3 Department of Microbiology, Immunology and Pathology, Colorado State University, Fort Collins, Colorado, United States of America; University of Medicine and Dentistry of New Jersey, United States of America

## Abstract

Defining the mechanisms of *Mycobacterium tuberculosis* (Mtb) persistence in the host macrophage and identifying mycobacterial factors responsible for it are keys to better understand tuberculosis pathogenesis. The emerging picture from ongoing studies of macrophage deactivation by Mtb suggests that ingested bacilli secrete various virulence determinants that alter phagosome biogenesis, leading to arrest of Mtb vacuole interaction with late endosomes and lysosomes. While most studies focused on Mtb interference with various regulators of the endosomal compartment, little attention was paid to mechanisms by which Mtb neutralizes early macrophage responses such as the NADPH oxidase (NOX2) dependent oxidative burst. Here we applied an antisense strategy to knock down Mtb nucleoside diphosphate kinase (Ndk) and obtained a stable mutant (Mtb Ndk-AS) that displayed attenuated intracellular survival along with reduced persistence in the lungs of infected mice. At the molecular level, pull-down experiments showed that Ndk binds to and inactivates the small GTPase Rac1 in the macrophage. This resulted in the exclusion of the Rac1 binding partner p67^phox^ from phagosomes containing Mtb or Ndk-coated latex beads. Exclusion of p67^phox^ was associated with a defect of both NOX2 assembly and production of reactive oxygen species (ROS) in response to wild type Mtb. In contrast, Mtb Ndk-AS, which lost the capacity to disrupt Rac1-p67^phox^ interaction, induced a strong ROS production. Given the established link between NOX2 activation and apoptosis, the proportion of Annexin V positive cells and levels of intracellular active caspase 3 were significantly higher in cells infected with Mtb Ndk-AS compared to wild type Mtb. Thus, knock down of Ndk converted Mtb into a pro-apoptotic mutant strain that has a phenotype of increased susceptibility to intracellular killing and reduced virulence *in vivo*. Taken together, our *in vitro* and *in vivo* data revealed that Ndk contributes significantly to Mtb virulence via attenuation of NADPH oxidase-mediated host innate immunity.

## Author Summary

Mycobacterium tuberculosis (Mtb) is a very successful intracellular pathogen that infects lung macrophages. Its resistance to intracellular killing has been linked to the development of pulmonary tuberculosis (TB) in humans. Thus, understanding the mechanism by which *Mycobacterium tuberculosis* (Mtb) persists in the host is a prerequisite for development of efficient strategies to control TB disease. We have previously shown that Mtb nucleoside diphosphate kinase (Ndk) contributes to phagosome maturation arrest via inactivation of Rab5 and Rab7. In this study, we show that Ndk also targets and inactivates the small GTPase Rac1, an essential component of the macrophage NADPH oxidase (NOX2) complex. Ndk-dependent inactivation of Rac1 was associated with reduced NOX2-mediated production of reactive oxygen species (ROS) and ROS-dependent apoptosis. Conversely, disruption of Ndk expression converted Mtb into a mutant strain that induces strong ROS and apoptosis responses. This phenotype was associated with reduced survival of Ndk mutant *in vitro* and *in vivo*. Altogether, our findings demonstrate that Ndk contributes significantly to mycobacterial virulence.

## Introduction

The ability of *Mycobacterium tuberculosis* (Mtb) to adapt and thrive intracellularly relies on a variety of strategies to alter mechanisms of the host innate immunity. In particular, interference with phagosome biogenesis was highlighted as a significant aspect of Mtb persistence and replication within the macrophage [1], [2]. How Mtb circumvents phagosomal acidity, bactericidal enzymes, and reactive oxygen species (ROS) remains a central question for many cellular microbiologists.

ROS are produced by the phagocyte NADPH oxidase (NOX2) complex and were classified 30 years ago as powerful microbicidal agents against many intracellular pathogens [3]. *In vivo* evidence for the contribution of NOX2 to the innate immunity arsenal was deduced from field observations of high susceptibility of chronic granulomatous disease patients (CGD) to opportunistic pathogens [4], [5]. Such observations were experimentally confirmed in mouse models of CGD [6], [7]. Recent years have seen a growing body of evidence to suggest a crucial role for ROS in the control of mycobacterial infections [7]. In particular, one group has recently identified Mtb *nuoG* as a potential virulence factor operating at the level of NOX2 by mechanisms yet to be defined [8].

The NOX2 complex consists of two constitutively associated transmembrane proteins, gp91^phox^ and gp22^phox^ and four cytosolic subunits: p40^phox^, p47^phox^, p67^phox^, and Rac1, a small GTPase [9]. Fully functional NOX2 requires membrane translocation of p40^phox^, p47^phox^, active Rac1 (GTP-bound form) and p67^phox^, and their assembly around gp91^phox^ and gp22^phox^ subunits [10]. NOX2 assembly leads to gp91^phox^ activation to generate superoxide through a redox chain by transferring electrons from cytosolic NADPH to phagosomal oxygen [9]. The production of superoxide is in turn converted into several other microbicidal molecules, such as hydrogen peroxide and hydroxyl radicals, along with peroxynitrite when combined with nitric oxide radicals [9]. While the role of NOX2 in innate immunity is well established, several reports suggested that it might act beyond the control of intracellular infections to trigger macrophage apoptosis [11], [12], a central event that paves the road to adaptive immunity [13]–[15].

Previous results from our laboratory identified Mtb nucleoside diphosphate kinase (Ndk) as a GTPase Activating Protein (GAP) acting on Rab5 and Rab7 GTPases, leading ultimately to reduced phagolysosome fusion [16], [17]. In the present study, we examined whether Ndk GAP activity extends to other GTPases, with a particular focus on Rho GTPases. We found that Mtb Ndk interacts specifically with Rac1 and inactivates it leading to inhibition of NOX2 assembly and activation in the macrophage. We also established a link between Ndk-dependent NOX2 attenuation and inhibition of apoptosis response to Mtb. Consistent with these findings, Ndk knock down significantly reduced Mtb survival *in vitro* and *in vivo*.

## Results

### Ndk contributes significantly to Mtb persistence

**Figure 1:**
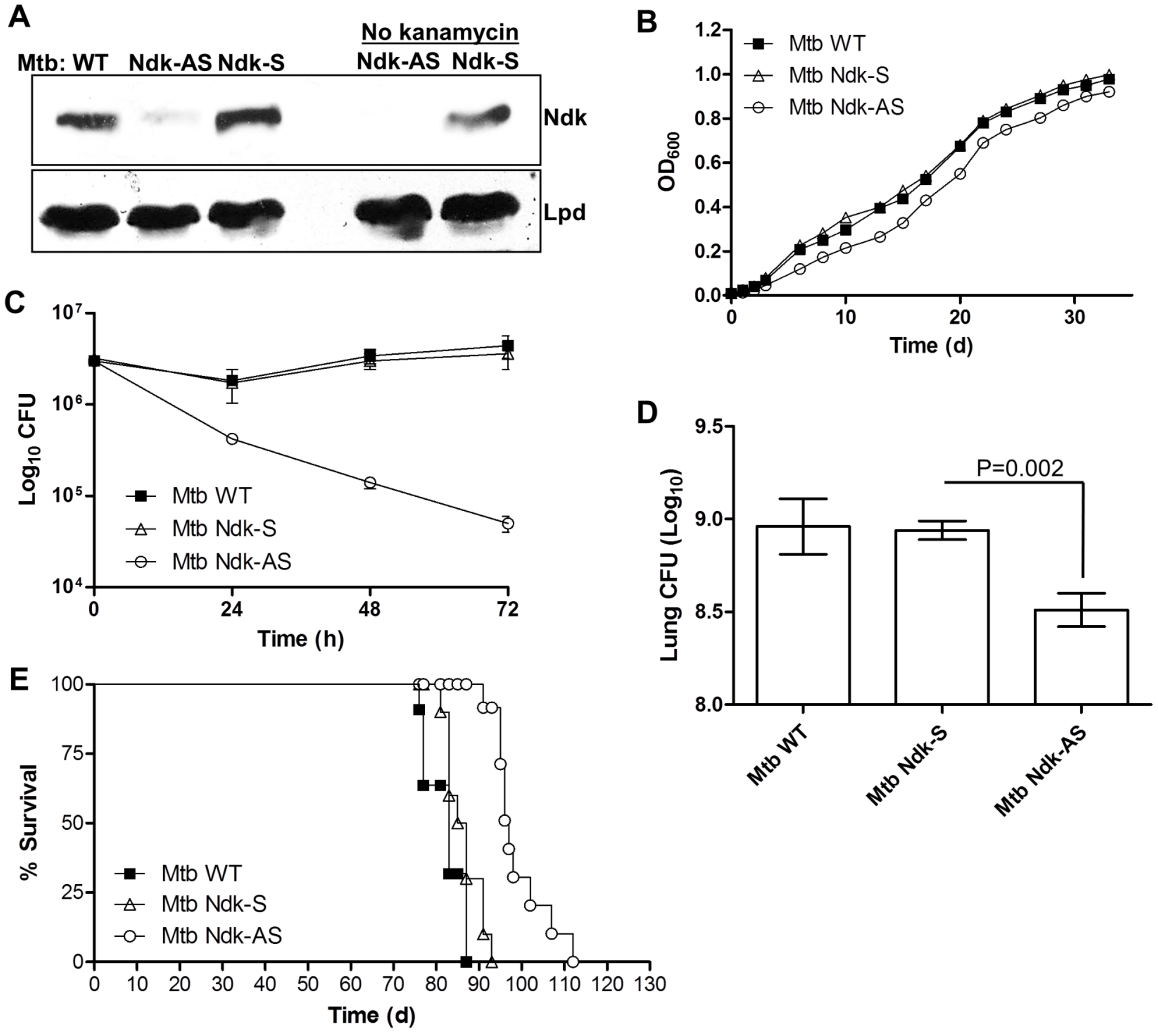
Knock down of Ndk attenuates the survival of Mtb in the host. **A**. Mtb Ndk-AS and Ndk-S strains were grown in the presence or absence of selection marker kanamycin then lysed along with wild type Mtb in 50 mM Tris, 5 mM EDTA, 0.6% SDS in the presence of protease inhibitors and 0.1 mm glass beads as described [17]. Samples were then subjected to SDS-PAGE and western blot analysis with Ndk and lipoamide dehydrogenase (Lpd) antibodies. The latter was used to confirm loading of equal amounts of proteins. **B**. Growth curves comparing wild type, Ndk-S and Ndk-AS strains expressed as absorbance at 600 nm. **C**. RAW 264.7 macrophages were infected with Mtb strains at a MOI of 10∶1 and then washed thrice 2 h post infection to remove extracellular bacteria. Cells were reincubated in media plus gentamicin (50 µg/ml) and subsequently lysed in 0.025% SDS at the indicated post-infection time points. Serial dilutions of recovered Mtb were then plated on solid 7H10 media supplemented with 10% OADC and CFU counts were performed after 3 weeks incubation at 37°C. Results (mean CFU ± SEM) correspond to 2 independent experiments. **D**. SCID mice (n = 10 per group) were infected with ∼150 bacteria by inhalation and then six weeks later the bacterial load in the lung was determined. **E**. SCID mice were infected subcutaneously with 10^6^ wild type, Ndk-S or Ndk-AS Mtb (n = 10 per group) and survival was monitored over four months.

We recently showed that mycobacterial Ndk plays an essential role in intracellular survival of the attenuated *M. bovis* BCG strain by a mechanism dependent on phagosome maturation arrest [17]. To examine whether Ndk also contributes to survival of virulent Mtb, we first attempted to generate an Ndk mutant in the Mtb strain H37Rv using various methods, including a gene disruption approach utilizing transducing mycobacteriophages [18]. Unfortunately, *ndkA* gene disruption affected severely the growth of bacteria. We therefore opted for protein knock down with mRNA antisense, the only approach developed so far to study essential genes in Mtb [19]. To do so, we transformed Mtb with the integrative vector pJAK1.A, previously designed by us [20], to express a stable full length antisense (or sense, control) mRNA sequence to *ndkA*. Thus, we generated a strain (Mtb Ndk-AS) in which Ndk protein expression was undetectable by western blot, even after many passages in the absence of the selection marker kanamycin, indicating a stable knock-down with the pJAK1.A vector (**Fig. 1A**). Fortunately, Mtb Ndk-AS displayed a similar growth profile to that of wild type Mtb and the control sense strain (Mtb Ndk-S) in standard culture media (**Fig. 1B**), as well as in the presence of oxidative stress (H_2_O_2_, **Fig. S1**). Thus potential fitness disadvantage that could be associated with genetic manipulation of Mtb are unlikely. Knock down of Ndk significantly affected Mtb survival in RAW 264.7 macrophages to the extent that at 72 h post infection, numbers of Mtb Ndk-AS dropped by 2 log colony-forming units (CFUs), relative to the wild type or Ndk-S strains (**Fig. 1C**). These findings suggested that the Ndk protein might contribute to Mtb virulence *in vivo*. Virulence during the early acute phase of infection is essentially controlled by innate immune responses and can be rapidly assessed in the SCID mouse model where innate immune responses are intact [21], [22]. In this regard, experiments of SCID mice infection by aerosol showed significant reduction (∼70%) of bacterial load in the lungs of Ndk-AS infected animals compared to those infected with wild type and Ndk-S strains (P = 0.002, unpaired t-test, **Fig. 1D**). Indicators of morbidity were apparent in the mice within 6 weeks with no significant difference between the three infection test groups (**Fig. S2**). However, when infected subcutaneously, time to death was extended to 12–15 weeks. Under these conditions, Kaplan Meier survival analysis clearly demonstrated that animals inoculated with Mtb Ndk-AS survived significantly longer (∼20 days, P<0.0001) than the control strain expressing Ndk sense mRNA, which caused the death of mice at similar rates seen in mice infected with the wild type strain (**Fig. 1E**). Taken together, these data demonstrated clearly that Ndk contributes to Mtb survival in the host through mechanisms that we have attempted to elucidate.

### Mtb Ndk binds to Rac1 GTPase

**Figure 2:**
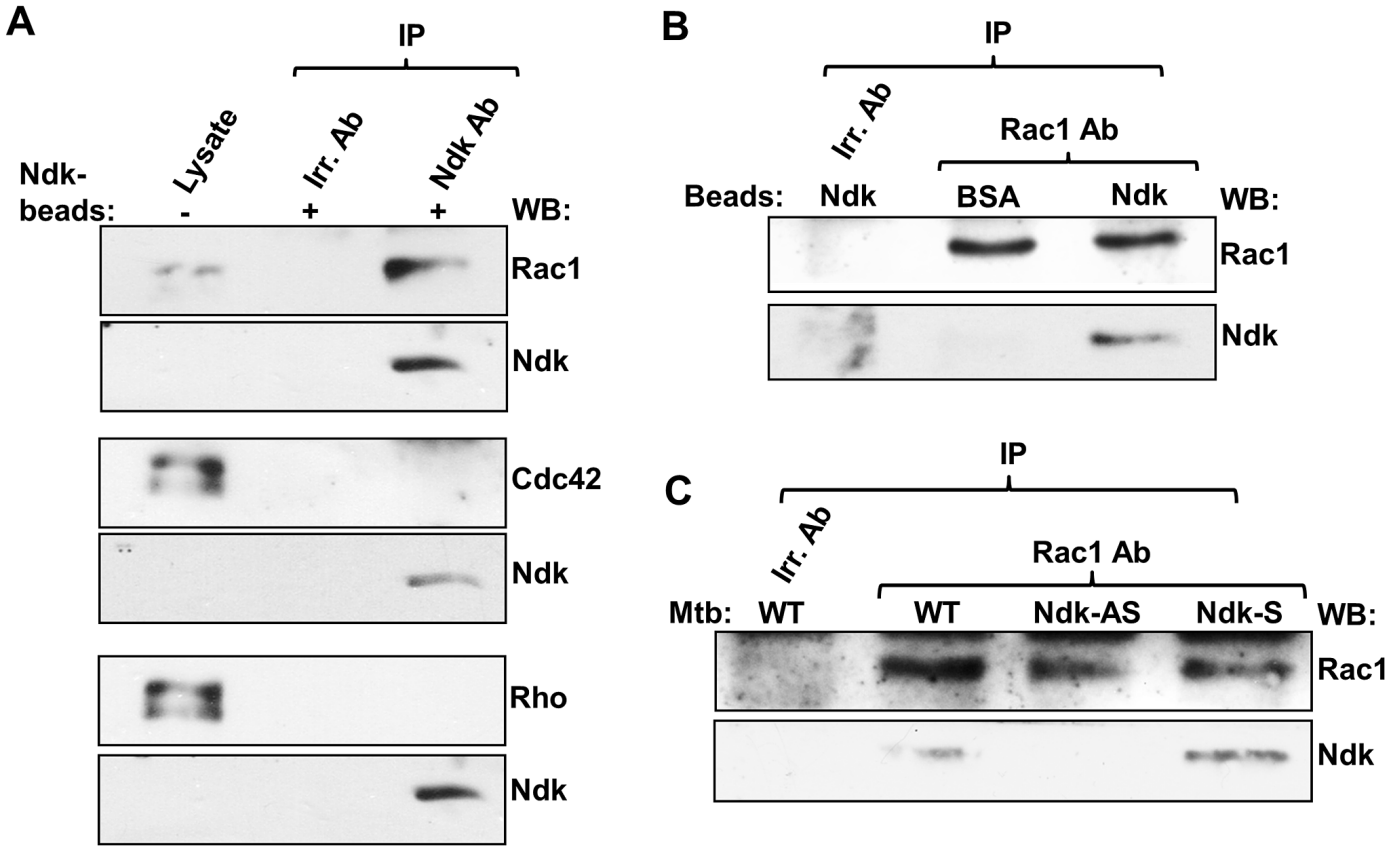
Mtb Ndk binds to Rac1 within the macrophage. **A**. Lysates from RAW macrophages ingesting Ndk-coated beads (MOI 5∶1) were incubated with Ndk or irrelevant (Irr.) antibody and protein A agarose beads. Pulled down Ndk and any associated proteins were analyzed along with whole cell lysate (first lanes) by western blot with antibodies to Rac1, Rho, or Cdc42. Rac1 (top set), but not Cdc42 (middle set) or Rho (bottom set), was found to be associated with Ndk intracellularly **B**. Reverse immunoprecipitation experiments: Macrophages were allowed to ingest Ndk or BSA (control) beads (MOI 5∶1), then subjected to similar immunoprecipitation experiments as in A., but using Rac1 or irrelevant antibody to pull down Rac1 and any associated proteins. **C**. Similar immunoprecipitation experiments as in B., but macrophages were infected with wild type, Ndk-AS or Ndk-S Mtb strains (MOI 20∶1) instead of coated beads. Top panel indicates total amount of pulled down Rac1, while bottom panel shows amount of Ndk associated to Rac1. Data are representative of three independent experiments.

Our recent findings that Ndk expresses GAP activity towards Rab5 and Rab7 [17] suggested that this activity might extend to other host GTPases. Therefore, we examined whether Ndk targets macrophage Rho GTPases, known to play essential roles in early events of innate immunity against intracellular pathogens [23], [24]. To do so, macrophages were allowed to ingest Ndk-coated latex beads and then cell lysates were subjected to immunoprecipitation with Ndk antibody. Proteins associated with Ndk were analyzed by western blot with Rac1, Rho, or Cdc42 antibodies. The results obtained showed that only Rac1 was interacting with Ndk within the macrophage (**Fig. 2A**). Rac1 binding to Ndk was further confirmed with reverse pull down experiments using Rac1 antibody and western blotting with Ndk antibody, which showed clearly a physical association between Mtb Ndk and Rac1 (**Fig. 2B**). Results obtained with coated latex beads clearly demonstrated the specificity of Ndk-Rac1 interactions within the macrophage. Accordingly, we then examined Ndk-Rac1 interaction in cells infected with the bacterium instead of beads and showed that Rac1 antibodies are able to pull-down Ndk-Rac1 complexes from cells infected with wild type and Ndk-S but not Ndk-AS Mtb (**Fig. 2C**)

### Mtb uses Ndk as GAP activity towards macrophage Rac1

**Figure 3:**
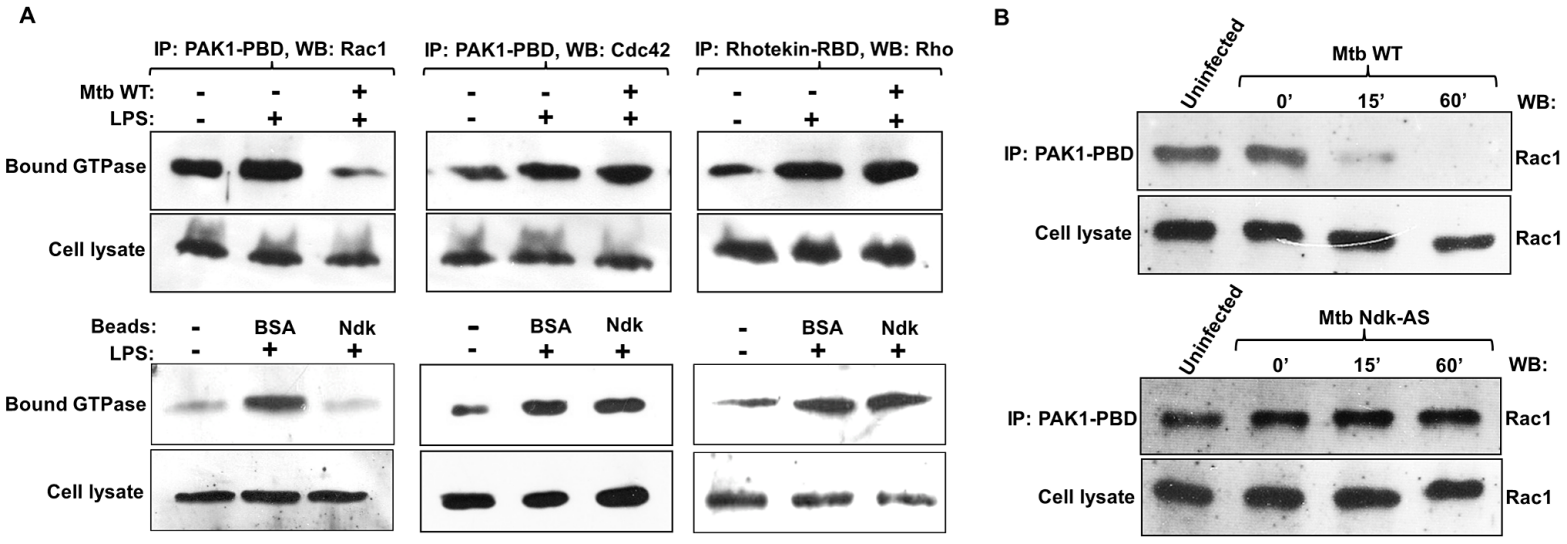
Mtb Inactivates Rac1 in infected macrophages. **A**. RAW macrophages were allowed to ingest Mtb wild type (WT) (MOI 20∶1) or coated latex beads (MOI 5∶1) then treated with LPS (200 ng/ml) for 15 min to activate Rho GTPases. Lysates were then prepared and examined for levels of active (GTP-bound) Rho GTPases by pull down with specific probes (Millipore kit) and western blot with Rac1, Rho, or Cdc42 antibodies (top panel). In the bottom panel, similar western blot analyses were applied to equal aliquots of untreated whole cell lysates to confirm that equal amounts of total Rho proteins were used in the pull down assay. **B**. RAW macrophages were infected with Mtb WT or Mtb Ndk-AS (MOI 20∶1) and cell lysates were assayed for Rac1 activation 0 min, 15 min, and 60 min post phagocytosis. Top panels indicate amount of active Rac1 detected in cell lysates and bottom panels indicates levels of Rac1 in untreated whole cell lysate aliquots. Data are representative of three independent experiments.

The results shown above (**Fig. 2**) suggested that Mtb Ndk must cross the phagosomal membrane towards the cytosol to bind to and inactivate Rac1. We first confirmed the hypothesis of cytosolic translocation of Ndk using i) confocal microscopy analysis, which showed diffused staining of Ndk distant from phagosomes containing wild type and Mtb Ndk-S but not from those containing Mtb Ndk-AS (**Fig. S3A**) and ii) immunogold staining and EM analysis, which clearly demonstrated that Mtb Ndk effectively crosses the phagosomal membrane toward the cytosol (**Fig. S3B**). We next examined the level of Rac1 activation in infected macrophages with pull down experiments using binding domain derived from Rac1 interacting protein (PAK-1 PBD), which interacts with Cdc42 as well. We also examined levels of Rho activation using Rho interacting protein (Rhotekin RBD). These binding domains interact only with GTP-bound forms of Rho GTPases [25]. Mtb infected RAW cells were exposed to LPS in order to activate the Rho GTPases, then cell lysates were examined for the amount of active Rac1, Rho, or Cdc42. Western blot analyses with Rac1, Cdc42 and Rho antibodies demonstrated that Mtb significantly inhibits the level of LPS-induced Rac1 activation (**Fig. 3A** **, top panel**). In contrast, Mtb had no apparent effect on Cdc42 and Rho activation. This GAP activity was also observed in macrophages ingesting Ndk-coated beads, as opposed to Mtb bacilli, demonstrating a specific Ndk GAP activity on Rac1 (**Fig. 3A** **, lower panel**).

To further examine Mtb effects on Rac1 and the phagosomal events it regulates, we performed a time-course Rac1 activation assay with macrophages infected by Mtb Ndk-AS and wild type Mtb. The results obtained showed a dramatic reduction of active Rac1 levels 15 min post infection and undetectable levels 1 h later in macrophages infected with wild type Mtb (**Fig. 3B** **, top panel**). In contrast, levels of active Rac1 remain unchanged in macrophages infected with Mtb Ndk-AS (**Fig. 3B** **, bottom panel**). Taken together, these data clearly demonstrated that Mtb Ndk expresses GAP activity on both basal and induced Rac1-GTP levels in the macrophage.

### Ndk disrupts phagosomal recruitment of p67^phox^

**Figure 4:**
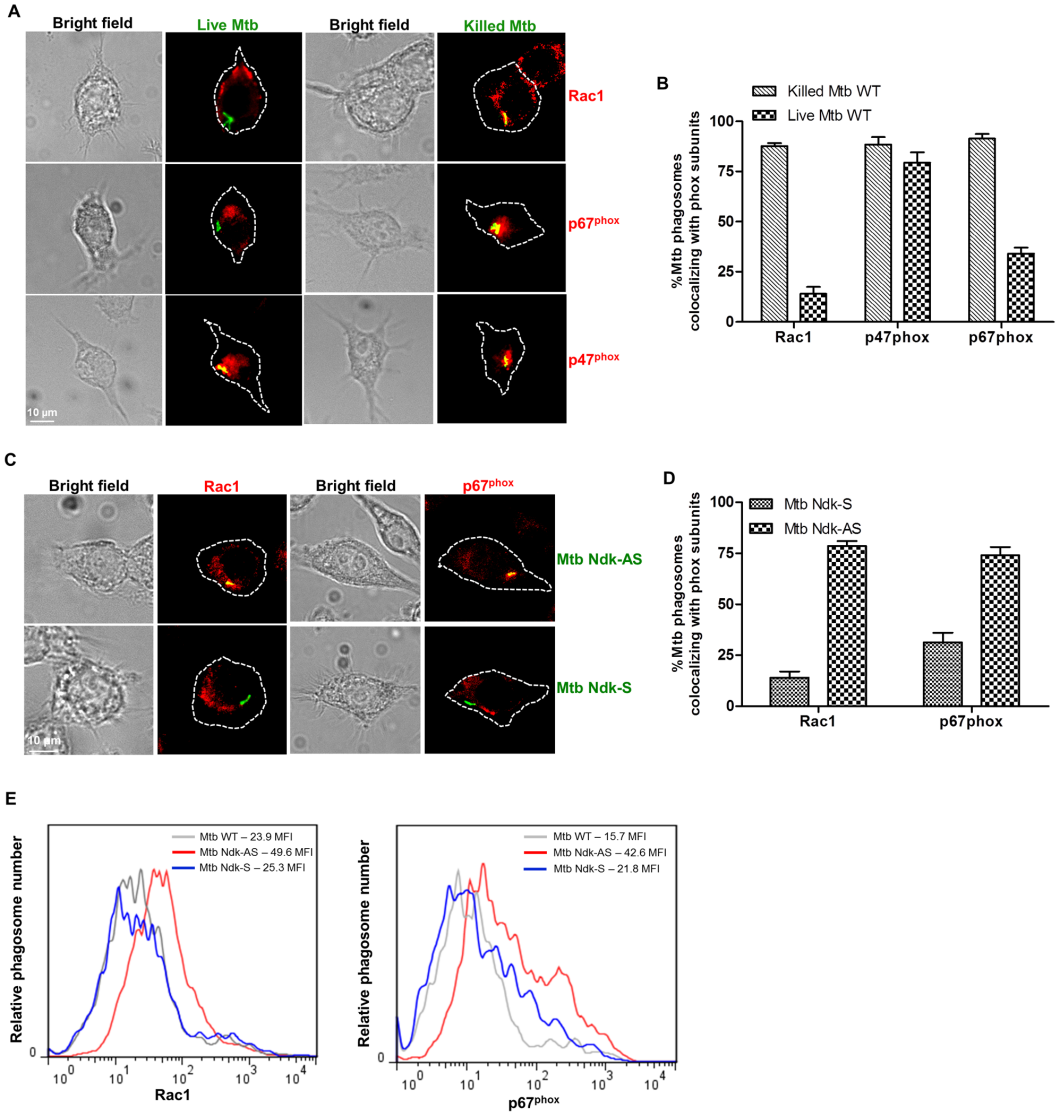
Decreased recruitment of Rac1 and p67^phox^ to Mtb phagosomes. **A**. RAW macrophages were infected with live or gentamicin killed (50 µg/ml, 1 h) Mtb expressing DsRed at MOI of 10∶1 for 1 h. Cells were then fixed/permeabilized and stained with Rac1, p47^phox^ or p67^phox^ antibodies and corresponding FITC-conjugated secondary antibodies. Cells infected with killed Mtb showed clear yellow signals indicating colocalization of the bacterial phagosome with all three phox subunits. Conversely, both Rac1 and p67^phox^, but not p47^phox^, were absent on phagosomes containing live bacteria. **B**. Quantification of the confocal data shown in panel A. **C**. Macrophages were infected with Ndk-AS and Ndk-S Mtb strains then immunostained as in A. The images show recruitment of Rac1 and p67^phox^ to phagosomes containing Ndk-AS but not Ndk-S bacteria. **D**. Quantification of the confocal data shown in panels C. Values in B and D are the mean ±SD of positive phagosomes in 50–80 cells from three independent experiments. **E**. Fluorescence histograms of phagosomal levels of Rac1 and p67^phox^ in infected macrophages obtained by FACS analysis approach described in **Fig. S4**.

Active Rac1 (GTP bound form) has been shown to translocate to early phagosomes in order to facilitate recruitment of its binding partner, the NOX2 subunit p67^phox^ [26], [27]. Given that Ndk expresses GAP activity towards Rac1 (GTP into GDP switch), we examined whether Mtb interferes with phagosomal recruitment of p67^phox^. We first applied intracellular staining and confocal microscopy to estimate the proportion of Rac1 and p67^phox^ positive phagosomes in Mtb-infected RAW cells. Results obtained (**Fig. 4A and 4B**) showed a substantial reduction of Rac1 and p67^phox^ positive phagosomes (13% and 29% respectively) in cells infected with live Mtb relative to those infected with killed Mtb (86% Rac1 and 88% p67^phox^ positive phagosomes, respectively). In contrast, recruitment of p47^phox^ to live Mtb phagosomes was comparable to that of phagosomes containing killed Mtb. To demonstrate that the NOX2 assembly defect is related to Ndk GAP activity, we applied similar confocal analyses to cells infected with Mtb Ndk-AS. The images (**Fig. 4C and 4D**) clearly showed a restoration of Rac1 and p67^phox^ recruitment to Mtb Ndk-AS containing phagosomes to a level similar to those observed in cells infected with killed Mtb (∼% and 72% positive phagosomes, respectively). As expected, much lower numbers of Rac1 and p67^phox^ positive phagosomes (13% and 30% respectively) were observed in cells infected with control strain Mtb Ndk-S.

As an alternative approach, a previously developed quantitative FACS analysis method [28] was used to assess the level of NOX2 components on individual phagosomes. To adapt this method to mycobacterial phagosomes, macrophage plasma membrane was stained with CellMask Deep Red (detectable by FL4 channel), and then cells were infected with Mtb strains expressing fluorescent DsRed protein (FL2). Following cell disruption, mycobacteria included in cell membrane-derived vacuoles (double FL2/FL4 positive events) were readily identified by flow cytometry analysis (**Fig. S4**). Phagosome preparations were then stained with Rac1 or p67^phox^ antibodies and FITC-conjugated secondary antibodies (FL1). Samples were subjected to flow cytometry analysis and mean fluorescence intensities (MFI) were deduced from fluorescence histograms. Results obtained (**Fig. 4E**) showed higher recruitment of Rac1 and p67^phox^ to phagosomes containing Mtb Ndk-AS (MFI: 49.6 and 42.6 respectively) relative to phagosomes containing Mtb Ndk-S (MFI: 25.3 and 21.8 respectively) or Mtb wild type (MFI: 23.9 and 15.7 respectively). To establish a direct link between Ndk GAP activity and defective NOX2 assembly, additional flow cytometry analyses were applied to phagosomes containing coated beads (**Fig. S5**) and showed a marked decrease of Rac1 and p67^phox^ recruitment to the Ndk bead phagosomes (MFI: 8.1 and 3.8 respectively) relative to control phagosomes containing BSA-beads (MFI: 14.6 and 7.3 respectively). Taken together, these findings showed for the first time that Mtb uses Ndk GAP activity to disrupt phagosomal assembly of NOX2 via interference with Rac1-dependent recruitment of p67^phox^.

### Mtb Ndk inhibits the macrophage oxidative burst

**Figure 5:**
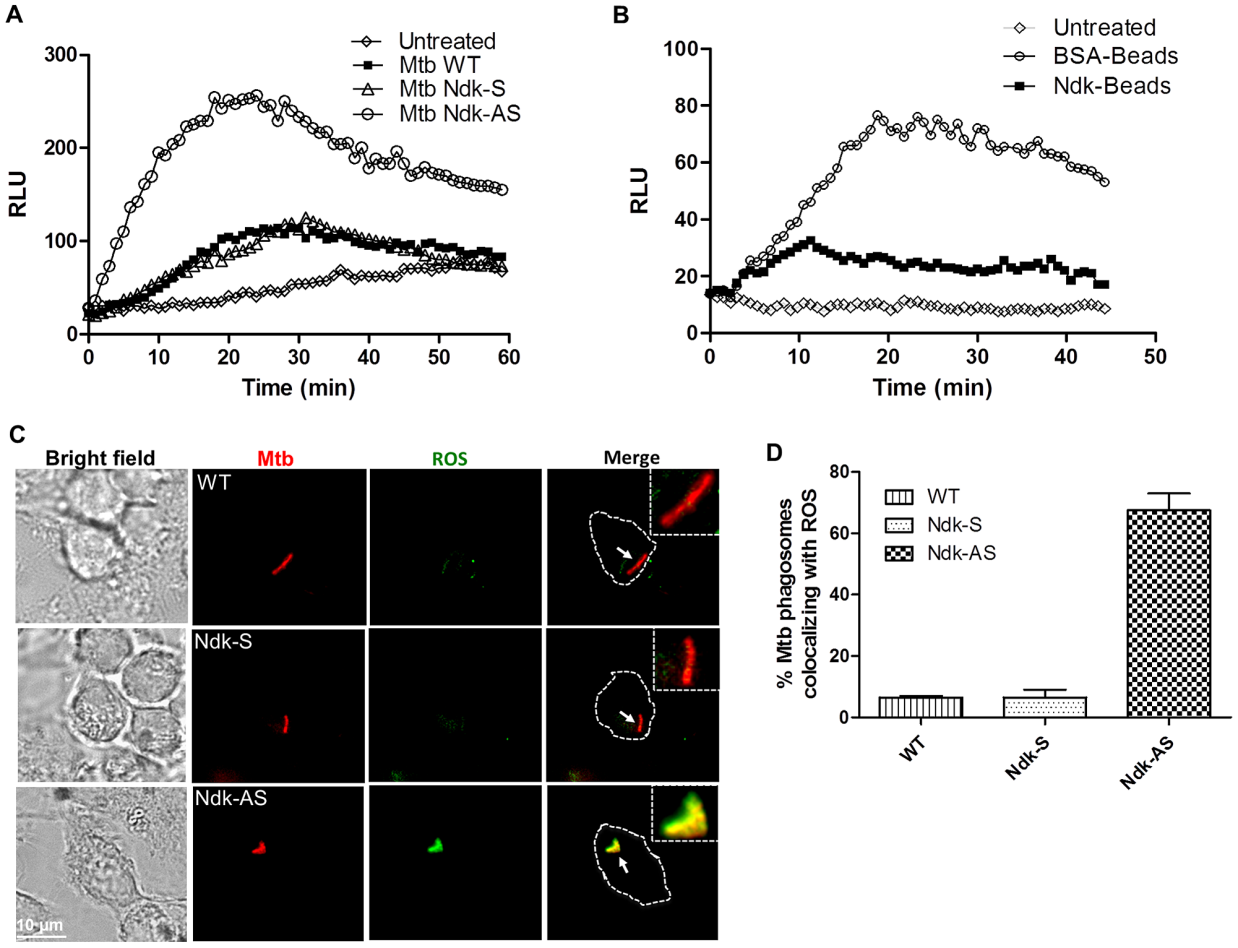
ROS production is inhibited in the presence of Mtb Ndk. **A**. BMDM were stimulated with 100 ng/ml LPS for 48 h then exposed to the indicated Mtb strains at MOI of 10∶1 (**A**) or coated latex beads at MOI of 5∶1 (**B**) in the presence of 50 µM luminol. Thereafter, luminescence was quantified by Tropix TR717 microplate reader. Results are expressed as Relative Luminescence Units (RLU) as a function of time **C**. RAW macrophages were loaded with 10 µM CM-DCFDA for 30 min at 37°C and subsequently infected by Mtb strains expressing DsRed (MOI of 10∶1) for 30 min. Thereafter, cells were washed and fixed with 2.5% PFA and mounted for analysis by confocal microscopy. Yellow signal indicates colocalization of phagosomes containing Mtb (red fluorescence) with oxidized CM-DCFDA (green fluorescence), indicative of ROS production. **D**. Mean ± SD of positive phagosomes observed in 50–80 cells from three independent experiments.

Previous findings that Rac1 and p67^phox^ subunits are essential for NOX2 assembly and activation of gp91^phox^ to generate superoxide [10] suggested that disruption of Rac1/p67^phox^ translocation to the phagosome by Ndk would affect NOX2-dependent ROS production. To verify this hypothesis, we applied a luminol-dependent chemiluminescence assay to assess ROS production in Mtb infected cells. Luminol is a membrane diffusible reagent commonly used for quantitative detection of superoxide anion radicals and hydrogen peroxide molecules. Bone marrow derived macrophages (BMDM) from C57BL/6 mice were infected with Mtb strains and assayed for kinetics of chemiluminescence production over a period of 60 min. Relative luminescence profiles obtained (**Fig. 5A**) revealed that cells infected with Mtb Ndk-AS induced significantly higher amounts of ROS production (peak value = 256 RLU) compared to those infected with wild type Mtb or Mtb Ndk-S (peak value ∼120 RLU). Thereafter, we confirmed the apparent inhibitory effect of Ndk with experiments using coated beads (**Fig. 5B**), which showed minor ROS response to Ndk beads (peak value = 32 RLU) relative to ROS production induced by BSA beads (peak value = 76 RLU). Additionally we applied confocal microscopy to visualize intracellular accumulation of ROS using CM-DCFDA, a cell-permeable probe that is non-fluorescent until oxidized within the cell. Thus, in RAW cells infected with Mtb Ndk-AS, the confocal images showed a strong colocalization of oxidized CM-DCFDA (green fluorescence) with bacterial phagosomes (red fluorescence) indicating accumulation of large amounts of ROS around Mtb Ndk-AS (**Fig. 5C and 5D**). Conversely, green signal was totally absent in cells infected with either wild type Mtb or Mtb Ndk-S. This effect of Ndk on ROS production was also reproduced when BMDM were used instead (**Fig. S6**).

Previous studies reported that mitogen-activated protein kinases (MAPKs) play an important role in the signaling pathway of NOX2 activation [29], [30]. To verify whether Ndk also interferes with MAPK activation, macrophages were allowed to ingest Ndk-beads or BSA-beads (control), and then stimulated with PMA or LPS to activate ERK1/2, and p38MAPK respectively. Cell lysates were then examined for the level of phospho-ERK1/2 and phospho-p38MAPK, which reflects kinase activation. The western blot results obtained (**Fig. S7**) did not reveal any changes in the levels of kinase phosphorylation in cells infected with Ndk-beads relative to those infected with BSA-beads. Therefore, Ndk effect on NOX2 is clearly independent of MAPK inhibition.

Collectively, these experiments demonstrated that the macrophage oxidative response to Mtb is marginal and that knock down of Ndk converts the bacterium into a potent inducer of the ROS response.

### Inhibition of NOX2 activity impairs apoptosis response to Mtb infection

**Figure 6:**
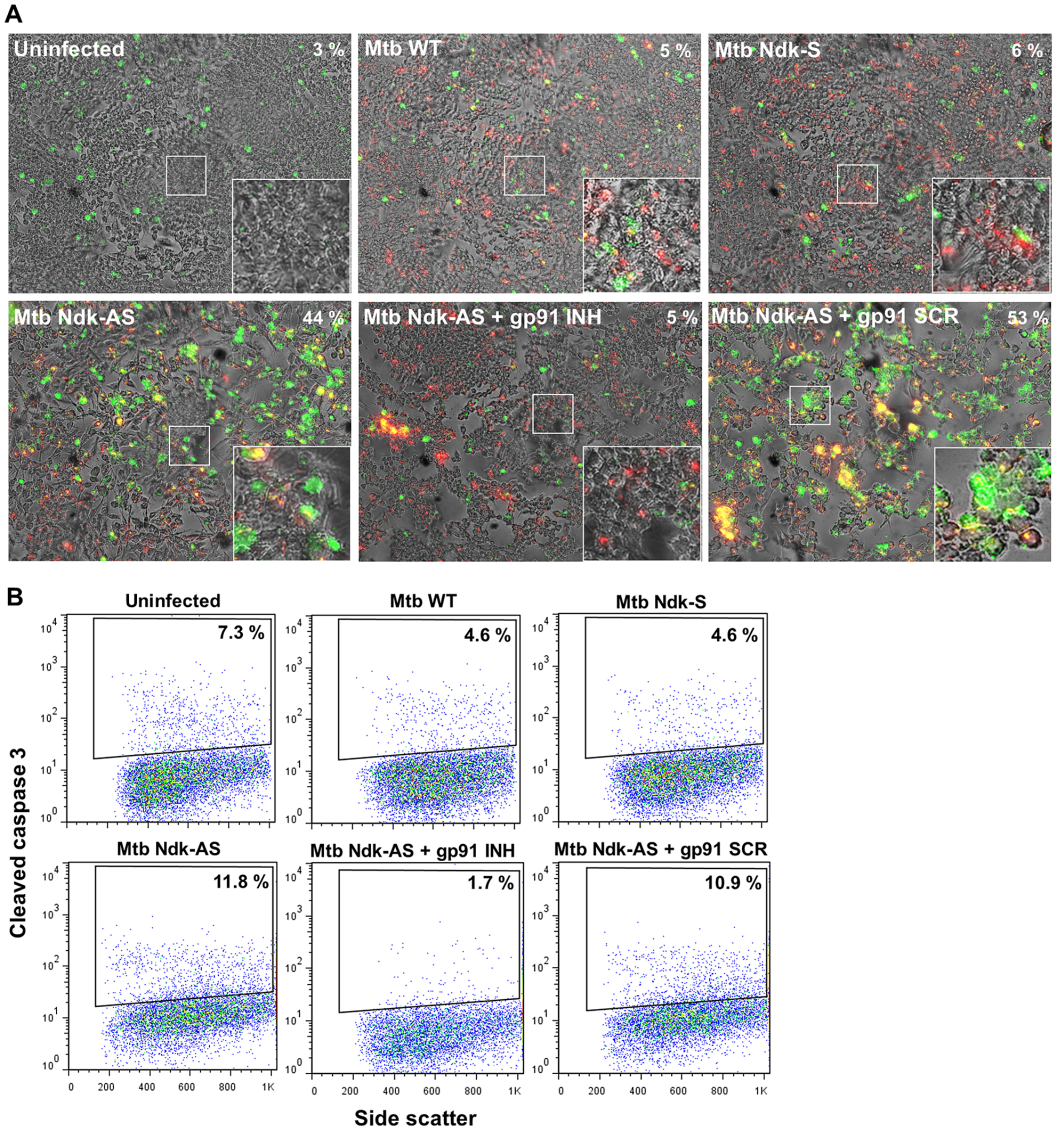
Mtb Ndk inhibits ROS dependent macrophage apoptosis. **A**. RAW macrophages were infected for 48 h with the indicated Mtb strains at MOI of 10∶1. To block ROS production, gp91^phox^ peptide inhibitor (gp91 INH) or its scrambled version (gp91 SCR) were added at 50 µM 1 h prior to infection by Mtb Ndk-AS. Cells were then stained with Alexa Fluor 488-Annexin V and analyzed by confocal microscopy. Images are a merge of bright field, red fluorescence (Mtb DsRed strains), and green fluorescence (Annexin V). Values in the top right corner indicate the percentage of apoptotic cells. **B**. Macrophages were infected as described in A., and then stained for cleaved caspase 3 as described in materials and methods. Cells with active caspase 3 were identified and quantified by FACS. Data are representative of two independent experiments.

Mtb is known to inhibit macrophage apoptosis [14], [15] by mechanisms yet to be clarified. Based upon previous findings that NOX2 activity might extend beyond intracellular killing to induce apoptosis [8], [14], we examined whether Mtb uses Ndk to disrupt the NOX2-apoptosis link. First, we applied Annexin V cell surface staining, a popular approach for detection of phosphatidylserine (PS) translocation to the extracellular membrane leaflet, which reflects early stages of apoptosis events [31]. Adherent RAW cells on coverslips were infected with Mtb strains for 48 h then stained with Alexa Fluor 488 conjugated Annexin V and examined by confocal microscopy. The images showed very low numbers of Annexin V positive cells in samples infected with wild type Mtb and Mtb Ndk-S (5% and 6% positive, respectively). In contrast, a higher number of Annexin V positive cells (44%) was observed in samples infected with Mtb Ndk-AS **(** **Fig. 6A** **, top panel**). To establish a direct link between ROS and apoptosis in infected cells, Annexin V staining was repeated on macrophages exposed to Mtb Ndk-AS in the presence of a specific gp91^phox^ peptide inhibitor (gp91 INH) or its control scrambled version (gp91 SCR) [32]. The results obtained showed clearly that gp91 INH, but not gp91 SCR, reversed completely Mtb Ndk-AS-induced PS translocation to the cell surface (5% Annexin V positive, **Fig. 6A**, **bottom panel**). The effect of the gp91^phox^ inhibitor was confirmed with experiments showing that gp91 INH completely inhibited ROS production in cells infected with Mtb Ndk-AS, which was normally elicited in the presence of gp91 SCR (**Fig. S8**). In a complementary series of experiments, we analyzed caspase 3 activation, which occurs during the final stages of apoptosis [33]. Thus, infected macrophages were subjected to intracellular staining with antibody to cleaved (i.e. active) caspase 3 and Alexa Fluor 647 conjugated secondary antibody, then analyzed by FACS. Results obtained (**Fig. 6B**) showed higher numbers of apoptotic macrophages in sample tests infected by Mtb Ndk-AS (11.8% positive events) relative to those infected with wild type Mtb or Mtb Ndk-S (∼4.6%). Not surprisingly, the wild type and Ndk-S strains inhibited the spontaneous apoptosis observed in control non-infected cells (7.3% positive cells). As expected, Mtb Ndk-AS-induced caspase 3 cleavage was abolished in the presence of the gp91^phox^ inhibitor.

It is well known that apoptosis is also induced by nitric oxide (NO) in mouse macrophages [34], [35]. Therefore, the effect of Ndk on macrophage apoptosis might be the result of simultaneous inhibition of ROS and NO production. To verify this possibility we examined IFN-γ-induced NO production in cells infected with Ndk-beads and BSA-beads (control) and the results deduced from the Griess assay (**Fig. S9**) demonstrated that Ndk has no effect on NO production.

Taken together, our data demonstrated that Mtb blocks macrophage apoptosis by a mechanism dependent, at least in part, on Ndk-mediated attenuation of NOX2 activity.

### Ndk knock down increases Mtb susceptibility to ROS-mediated intracellular killing

**Figure 7:**
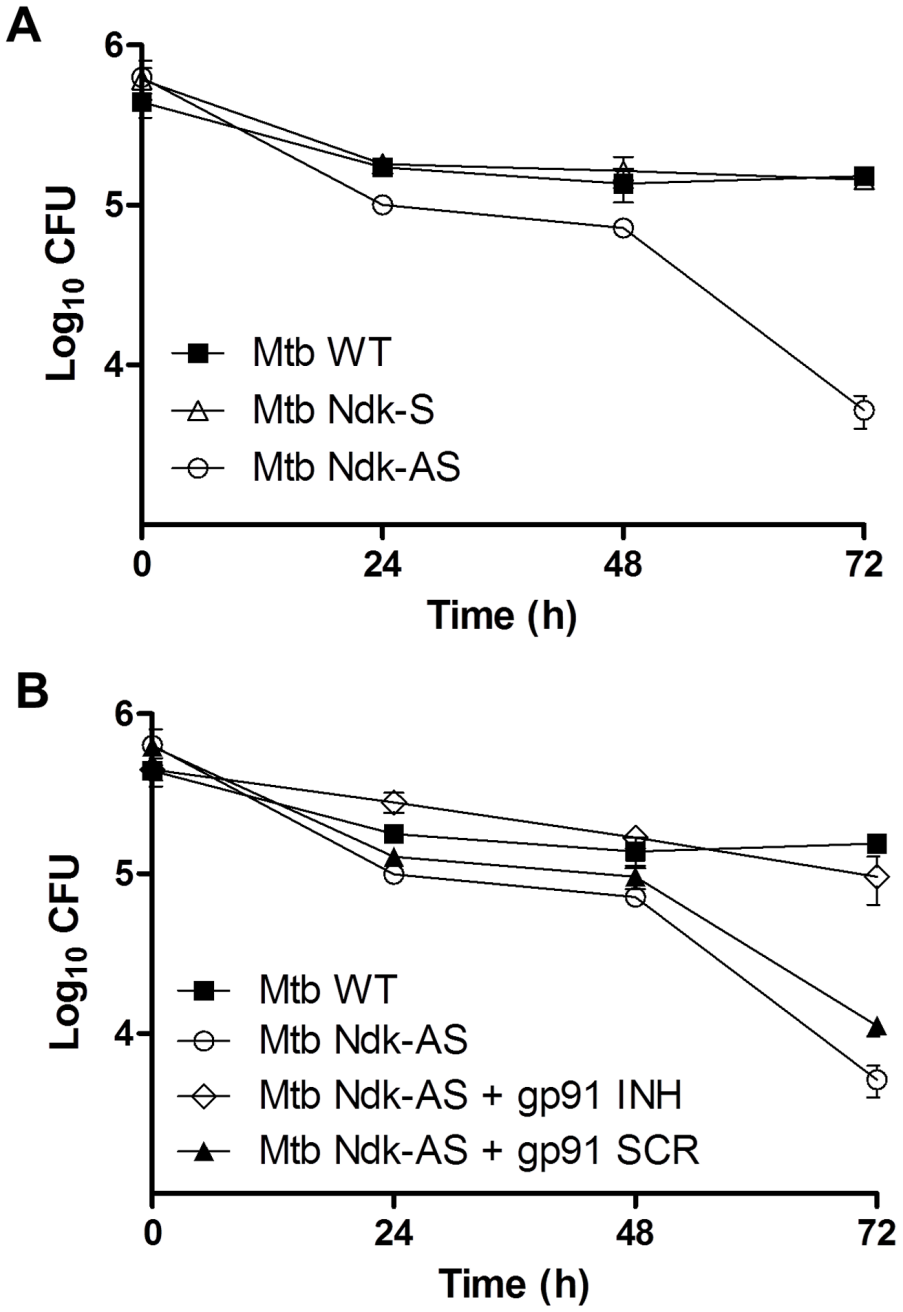
Inhibition of ROS production prevents intracellular killing of Mtb Ndk-AS. **A**. BMDMs (5×10^5^/well) were infected with the indicated Mtb strains (MOI of 10∶1) and washed thrice 2 h post infection to remove extracellular bacteria. Thereafter, cells were reincubated in media plus gentamicin (50 µg/ml) and lysed in 0.025% SDS at the indicated post-infection time points. Serial dilutions of recovered Mtb were then plated on solid 7H10 media supplemented with 10% OADC and CFU counts were performed after 3 weeks incubation at 37°C. **B**. Similar experiment as in A., in the presence or absence of 50 µM gp91^phox^ inhibitor peptide (or its scrambled version) added 1 h prior to infection with Mtb Ndk-AS. Results (mean CFU ± SEM) correspond to 2 independent experiments.

Results presented above (**Fig. 6**) together with initial experiments showing attenuated Mtb Ndk-AS survival in RAW macrophages (**Fig. 1**) suggested that Ndk-mediated inhibition of ROS reduces the macrophage killing capability. To verify this hypothesis, we repeated the survival assay using primary murine macrophages in which ROS production was blocked with gp91 INH. At 72 h post-infection, control experiments showed a significant reduction (∼1.5 Log_10_) in CFU counts when infecting with Mtb Ndk-AS relative to wild type or Ndk-S (**Fig. 7A**). However, in the presence of gp91 INH, Ndk-AS survival was restored to a level comparable to that of wild type Mtb at every time point measured (**Fig. 7B**). These observations were validated with assays in the presence of control scrambled peptide, which did not affect Ndk-AS survival. Taken together, these experiments clearly demonstrated that down modulation of ROS production by Ndk contributes significantly to Mtb persistence in the macrophage.

## Discussion

Previous studies from this laboratory showed that Mtb Ndk exhibits GAP activity towards Rab5 and Rab7 leading ultimately to diminished phagosomal recruitment of their respective effectors EEA1 and RILP [16], [17]. Defective recruitment of EEA1 and RILP correlated with reduced maturation of phagosomes containing Ndk mutant *M. bovis* BCG or Ndk-coated beads [17]. In the current study, we demonstrate that Ndk further enhances Mtb virulence by additional GAP activity towards the Rho GTPase Rac1. We provided direct evidence that Ndk blocks phagosomal recruitment for both Rac1 and its partner molecule p67^phox^ leading ultimately to inhibition of NOX2-mediated ROS production and ROS-mediated apoptosis. A link between Mtb GAP activities and virulence was established with the observation of reduced survival of Ndk mutant Mtb *in vitro* and *in vivo*.

Ndk is a ubiquitous small protein (∼15 kDa) found in virtually all organisms, from eukaryotes to prokaryotes. In Mtb, Ndk catalyzes the production of nucleoside triphosphates as precursors for RNA, DNA and polysaccharide synthesis, which are critical for normal bacterial physiology [36]. This possibly explains why our attempts to knock out the *ndkA* gene in Mtb were unsuccessful, suggesting that Ndk is probably essential for Mtb growth. Contrasting with this hypothesis, an effort to comprehensively identify all genes required for Mtb growth using the transposon site hybridization (TraSH) technique suggested that the Ndk gene is not essential for Mtb growth [37]. However, as cautioned by the authors of that study, TraSH is simply a screening tool and therefore cannot provide a definitive conclusion about gene essentiality. Indeed, several genes known to be essential for Mtb growth, such as *ideR* [38], *rmlD* [39] and *whiB2* [40] have not been identified as essential by the TraSH approach. Thus, whether or not Ndk is essential is a research question that is still open for further investigation and remains beyond the scope of our current study, which focused instead on deciphering the mechanisms by which Ndk promotes Mtb survival in the host.

Mycobacterial Ndk has been shown to interact with and inactivate recombinant Rho, Cdc42 and Rac1 proteins [41]. Here we found that within the macrophage, both Mtb and recombinant Ndk (delivered on the surface of latex beads) interact with and inactivate native Rac1, but not Rho or Cdc42. This suggests that results obtained from cell free systems do not always reflect host-pathogen interactions in the whole cell system. Not surprisingly, this type of discrepancy has been observed with other pathogens that use GAP activities as a mechanism of virulence. For instance, secreted YopE from *Yersinia*, and SptP from *Salmonella* were shown to have GAP activity towards all three Rho GTPases extracellularly. However, YopE acts only on Rac1 and RhoA, [42] whereas SptP inactivates Rac1 and Cdc42, but not RhoA, [43] in cultured cells. In the case of *Yersinia*, a recent study established a direct link between YopE-mediated inactivation of Rac1 and inhibition of ROS production [44] consistent with our findings that selective Ndk GAP activity towards Rac1 is sufficient to block ROS production in the macrophage. Inhibition of ROS production in nascent phagosomes has also been reported in macrophages infected with the protozoan *Leishmania*, an intracellular pathogen that is structurally and metabolically distinct from Mtb, which interferes with NOX2 by a mechanism independent of GAP activities [45]. Indeed, *Leishmania* was shown to use its abundant surface lipophosphoglycan to restrict phagosomal recruitment of both p47^phox^ and p67^phox^ but not Rac1. Conversely, our study showed that Ndk disrupts the recruitment of Rac1 and its binding partner p67^phox^ but not p47^phox^. The p47^phox^ subunit contains a PRR (Prolin Rich Region) at its C-terminus that binds with high affinity to the C-terminal SH3 domain of p67^phox^ in the cytosol [46], [47]. It also contains a PH (Pleckstrin Homology) domain that interacts specifically with membrane PI[3,4]P2 and phosphatidic acid [48]. While the tail-to-tail association of p47^phox^ and p67^phox^ plays a crucial role in NOX2 assembly [27], recent studies showed that it is rapidly disrupted after membrane translocation [49]. Therefore dissociated p47^phox^ and p67^phox^ would remain separately attached to the phagosome via membrane lipids and Rac1 respectively. This phagosomal configuration of NOX2 subunits is consistent with the specific dissociation of p67^phox^ from Mtb phagosomes as a result of Ndk-mediated Rac1 inactivation.

The overall emerging picture from ongoing studies of phagosome remodelling by Mtb suggests that more than one virulence determinant might act in concert to modulate a single event of phagosome biogenesis. For instance, the cell wall glycolipid lipoarabinomannan, which blocks the Ca^2+^ signaling pathway [50], synergizes with the acid phosphatase SapM, which hydrolyzes PI[3]P [51], to abolish phagosome maturation processes that are dependent on recruitment of EEA1. Such a synergism appears to also be the case for mycobacterial interference with NOX2 activity on the phagosomal membrane. Indeed, a recent study showed that the NuoG subunit of the type I NADH dehydrogenase also promotes Mtb interference with NOX2 activity, as evidenced by increased levels of ROS on Mtb Δ*nuoG* phagosomes [8]. However, the finding that NuoG is not secreted raises a question about the mechanistic connection between distant NuoG, contained within the bacterial cytosol, and NOX2 components on the cytosolic face of the phagosome membrane. Conversely several different groups have shown that Mtb Ndk is secreted [52]–[54], suggesting that Ndk could translocate to the cytosolic surface of the vacuole where it interacts with Rac1. In fact, our EM and confocal data revealed that secreted Ndk crosses the phagosomal membrane towards the cytosol. Consistent with these findings, previous studies showed that live Mtb exports a variety of proteins and glycolipids intracellularly [55]–[57], and that many of them cross the phagosomal membrane towards the host cell cytosol to interact with and inhibit critical regulators of phagosome biogenesis [51], [56], [58]. A possible mechanism for the cytosolic translocation of mycobacterial products is the generation of a semi-porous phagosome membrane by the Mtb ESX-1 secretion system [59], which was also shown to play an essential role in Mtb escape from the phagosome in later stages of infection [60], [61]. Therefore it is possible that the ESX-1 secretion system also mediates translocation of Ndk to the cytosol.

A highly relevant finding from the present study is that Ndk knock down converted virulent Mtb into an attenuated strain that lost resistance to the hostile environment of the host cell. Indeed, Mtb Ndk-AS infected cells were able to generate NOX2-dependent ROS production and also to undergo apoptosis thus ensuring maximal restriction of bacterial proliferation. In contrast, virulent Mtb strains were shown to down-modulate apoptosis in favor of necrosis [62], [63], which releases viable intracellular bacilli for further spreading of the infection and tissue damage during active tuberculosis disease. The link between ROS production, apoptosis and intracellular killing demonstrated in our study is consistent with earlier findings that intracellular oxidative stress induces phosphatidylserine externalization and increased caspase 3 activity [64], [65], and that apoptosis induced by the Fas ligand attenuates Mtb survival within the macrophage [66]. In addition to restricting the niche for mycobacterial replication, macrophage apoptosis contributes indirectly to the initiation of adaptive immunity mediated by dendritic cells. Indeed, infected macrophages undergoing apoptosis shed vesicles loaded with bacterial material (or apoptotic blebs) that prime dendritic cells for enhanced presentation of mycobacterial antigen to T cells [13]–[15].

In summary, while the role of Ndk in physiological processes has been intensively investigated, its contribution to Mtb pathogenesis has not been previously addressed. Our recent findings and current investigations have extended the knowledge of the biological effects of Ndk, to include inactivation of GTPase effector functions in the macrophage, therefore highlighting a novel strategy used by Mtb to circumvent host innate immunity

## Materials and Methods

### Reagents and chemicals

DMEM, Fetal calf serum (FCS), and HBSS were purchased from Gibco Laboratories (Burlington, ON, Canada). Luminol, CM-DCFDA, Annexin V-488, and CellMask Deep Red, were purchased from Invitrogen (Burlington, ON, Canada). Endotoxin-free culture reagents were from StemCell Technologies (Vancouver, BC, Canada). Protease inhibitor mixture, PMSF, and trypsin-EDTA were purchased from Sigma-Aldrich (St. Louis, MO). Protein A-agarose beads were from Bio-Rad laboratories (Hercules, CA). Aldehyde/sulfate latex beads (diameter, 4 µm) were obtained from Interfacial Dynamics (Portland, OR). gp91^phox^ inhibitor peptide and its scrambled version [34] were synthesized by GenScript (Piscataway, NJ).

### Antibodies

Rac1, RhoA, Cdc42 antibodies were purchased from Millipore (Temecula, CA). p67^phox^ antibody was purchased from BD Transduction Laboratories (Mississauga, ON, Canada) and 47^phox^ antibody was purchased from Santa Cruz Biotechnology (Santa Cruz, CA). Cleaved caspase-3 (Asp175) antibody was purchased from Cell Signaling (Danvers, MA). Alexa Fluor 647-conjugated anti-rabbit IgG was purchased from Invitrogen. FITC-conjugated anti-rabbit and anti-mouse IgG were purchased from Sigma Aldrich.

### Bacteria

*M. tuberculosis* H37Rv and its derivative strains were grown in Middlebrook 7H9 broth (BD Diagnostic Systems, Mississauga, ON, Canada) supplemented with 10% (v/v) OADC (oleic acid, albumin and dextrose solution; BD Diagnostic Systems) and 0.05% (v/v) Tween 80 (Sigma-Aldrich) at 37°C on standing culture. Mtb Ndk-AS and Ndk-S were generated using our integrative pJAK1.A plasmid (selection marker, kanamycin [17] encoding the full length *ndkA* gene in sense and anti-sense orientations as described [17]. To generate red-fluorescent bacteria, Ndk-S, Ndk-AS, and wild type Mtb strains were transformed with pSMT3 vector (selection marker, hygromycin) encoding the DsRed protein as described [16].

### Cell culture and infection

RAW 264.7 macrophages (ATCC, Manassas, VA) were maintained in 10 cm diameter culture dishes (Corning Inc., Corning, NY) at a density of ∼10^5^ per cm^2^ in Endotoxin-free DMEM containing 5% FCS and 1% each of L-glutamine, penicillin-streptomycin mixture, HEPES, non-essential amino acids (100× solution, StemCell). Bone marrow derived macrophages (BMDM) were obtained by flushing out femurs and tibias of 6–8 week old female C57BL/6 (Jackson Laboratory, Sacramento, CA) according to protocols approved by the University of British Columbia Animal Care and Use Committees. Cells were then maintained in complete DMEM containing 10 ng/mL M-CSF for 6 days. For macrophage infection, bacteria in mid-log phase were harvested by 5 min centrifugation at 8,000× *g*. They were subsequently washed twice with 7H9 plus 0.05% tween and passed several times through 25 gauge needles to break bacterial clumps. Thereafter, numbers of bacteria were normalized by optical density (OD_600_ 1.0 = 3×10^8^ bacteria/ml) and adjusted for the desired MOI. Macrophages were then exposed to Mtb strains in complete media without antibiotic for 2 h at 37°C and then washed thrice to remove extracellular bacteria. Cells were reincubated in complete media plus gentamicin (50 ug/ml) for the desired time periods.

### Animal studies

Groups of 4- to 6-week old female Fox Chase SCID mice (CB17/Icr-Prkdcscid/IcrCrl) were infected with ∼150 bacteria by inhalation using a Glas-Col inhalation exposure system (Terre Haute, IN). Two mice from each group were processed on day 1 following infection to confirm bacterial deposition in the lung. Remaining animals were monitored for signs of morbidity. Mice were then euthanized and the bacterial load (CFUs) in the lung was determined. Organs were homogenized and serial dilutions plated in duplicate on nutrient 7H10 agar. In other experiments, SCID mice were injected subcutaneously in the scruff of the neck with 10^6^ Mtb strains and then monitored for morbidity over a period of ∼15 weeks.

All animals were maintained in accordance with protocols approved by the Animal Care and Use Committees at the University of British Columbia. Experiments were approved by the Animal Care and Usage Committees and performed according to the Canadian Council on Animal Care Guidelines. The animal assurance welfare number is A11-0247.

### Preparation of recombinant Ndk and coating of latex microspheres

Ndk was expressed as a C-terminal 6×His tagged fusion protein in *E. coli* strain BL21 and purified using Ni-NTA purification resin (Qiagen) as described [17]. The purity of eluted Ndk was confirmed by SDS-PAGE and Coomassie Blue staining (**Fig. S10A**). Rabbit Ndk antibody was generated by GenScript, using KLH conjugated ELASQHYAEHEGK peptide fragment corresponding to amino acids 44 to 56 of Mtb Ndk. The specificity of Ndk antibody is shown in **Fig. S10B**. Ndk or the control BSA were non-covalently linked to latex beads as described [17].

### Fluorescence microscopy

Coverslips were mounted on microscope slides and examined by digital confocal microscopy as described [17].

### Immunoelectron microscopy

Immunogold staining was conducted at the EM Facility of the James Hogg Research Centre (Saint Paul Hospital, Vancouver, BC, Canada). In brief, Mtb-infected macrophages were fixed with 4% paraformaldehyde, embedded in 4% low melting point agarose and dehydrated in ethanol. Samples were then transferred to LR White resin. After polymerization at 50°C, 60 nm sections were cut with a Leica EM UC6 microtome (Leica Microsystems, Switzerland) and collected on nickel grids. Samples were labelled with Ndk antibodies then F(ab′)_2_ of ultra-small goat-anti-rabbit IgG (Electron Microscopy Sciences, Hatfield, PA). Sections were then post-fixed in 2% glutaraldehyde and subjected to silver enhancement for gold labeling with Silver R-Gent SE-EM (Aurion, Wageningen, Netherlands). Samples were then washed in distilled water, stained in 2% uranyl acetate, washed again, air dried and examined with a Tecnai 12 electron microscope (FEI Company, Hillsboro, OR).

### Rho GTPase activation assay

Confluent RAW cells seeded on 6 cm plates were infected by Mtb strains for 1 h at a MOI of 20∶1. Thereafter, cells were incubated in the presence of 200 ng/ml LPS for 15 min to induce Rho GTPase activation. Subsequently, cells were lysed in cold buffer containing 30 mM HEPES (pH 7.2), 100 mM NaCl, 10% glycerol, 1% Triton X100, 1 mM EDTA, 10 mM MgCl_2_, and 1 mM PMSF. Soluble protein fractions were analyzed for levels active Rho-GTPases by using a Rac/Rho/Cdc42 activation assay kit (Millipore).

### ROS detection assay

Macrophages were cultured in complete DMEM in 96 well white plates (Corning) at 10^5^ per well. Prior to ROS assay, cell media was replaced with DMEM without phenol red and luminol was added to a final concentration of 50 µM. Wells were then infected with Mtb strains (MOI 10∶1) or coated beads (MOI 5∶1). Thereafter, plates were loaded into a Tropix TR717 microplate luminometer (Applied Biosystems, Bedford, MA) adjusted to 37°C and relative luminescence was then measured at 60 sec intervals over 60 min. Intracellular detection of ROS was achieved by incubating adherent macrophages to cover slips with 10 µM CM-DCFDA for 30 min at 37°C prior to infection with Mtb strains expressing DsRed. Cells were then analyzed by confocal microscopy.

BMDMs, but not RAW cells, were primed with LPS (100 ng/ml, 48 h) prior to ROS assays because expression of fully functional NOX2 complex in BMDMs requires priming with LPS or TNFα [67], [68].

### Apoptosis assays

Adherent RAW cells on coverslips were infected with Mtb strains. At 48 h post phagocytosis, cells were washed twice with cold PBS and then incubated in Alexa Fluor 488 Annexin V (1∶20, Invitrogen) in staining buffer containing 10 mM HEPES (pH 7.4), 140 mM NaCl, and 2.5 mM CaCl_2_, for 20 min at room temperature. Coverslips were then analyzed by confocal microscopy. Alternatively, infected cells were scraped and fixed in PBS plus 2% paraformaldehyde for 15 min at room temperature. Cells were then washed with PBS and incubated with anti-cleaved caspase-3 (1∶250) in permeabilization buffer (0.1% Triton X100 and 1% BSA in PBS) for 20 min at room temperature. Thereafter, cells were washed and stained with Alexa Fluor 647-conjugated goat anti-rabbit IgG (1∶200) for 20 min at room temperature, washed again and analyzed by FACS.

## Supporting Information

Figure S1 Equal number of exponentially growing bacteria (∼10^7/^ml) were exposed to the indicated concentrations of H_2_O_2_ for 4 h. Thereafter 100 µl aliquots were plated in duplicates on 7H10 (WT Mtb) or 7H10 plus kanamycin (Mtb Ndk-S and -AS) for 3 weeks. Results are expressed as Log_10_ of CFUs.
(TIF)

Figure S2 Fox Chase SCID mice (n = 10 per group) were infected with ∼150 bacteria by inhalation and survival was monitored over six weeks.
(TIF)

Figure S3 RAW 264.7 macrophages were infected with Mtb strains expressing DsRed (red fluorescent) at a MOI of 10∶1 and then washed thrice 2 h post infection to remove extracellular bacteria, then reincubated for additional 4 h. **A**) Cells were fixed/permeabilized, stained with rabbit Ndk antibodies and FITC-conjugated anti-rabbit IgG (green fluorescence), then examined by confocal microscopy. The images shown are the merge of green and red signals. The yellow signal reflects detection of Ndk (green) within the bacteria (red). Such signal is strong in cells infected with wild type and Ndk-S as both produce substantial levels of Ndk, but very weak in cells infected with Ndk-AS strain, which produces very little Ndk. White arrows indicate Ndk trafficking beyond bacterial phagosomes (green signal alone) in cells infected with wild type (WT) and Ndk-S strains. **B**) Macrophages infected with wild type Mtb or Mtb Ndk-AS were fixed with 4% paraformaldehyde, embedded in LR White resin then cut (60 nm sections) with a Leica EM UC6 microtome. Sections were collected on nickel grids and labelled with Ndk antibodies then F(ab′)_2_ of ultra-small goat-anti-rabbit IgG. Sections were then post-fixed in 2% glutaraldehyde and subjected to silver enhancement for gold labeling with Silver R-Gent SE-EM. Samples were then washed air dried and examined with a Tecnai 12 electron microscope. Arrowheads indicate Ndk localized on the phagosomal membrane and full arrows indicate translocated Ndk to the cytosol. Background signal was absent in control sections stained with secondary antibody alone.
(TIF)

Figure S4 FACS analysis of Mtb phagosomes. Macrophage cell surface is labelled with CellMask Deep Red (detectable by FL4 channel) at 0.2 µg/ml for 5 min at 37°C prior to infection with DsRed mycobacteria (FL2). Then cells are treated with Trypsin-EDTA to remove non-ingested but partially attached bacteria. Thereafter, cells are homogenized in 20 mM HEPES buffer, pH 7.4 containing at, 0.25% sucrose, 0.1% BSA, and 0.5 mM EGTA. Homogenates were then centrifuged at 300× *g* for 2 min to remove nuclei and intact cells and the upper fractions were collected and centrifuged at 3,200× *g* for 10 min at 4°C. The pellets correspond to crude phagosome preparations where bacteria included in cell membrane-derived vacuoles (double FL2/FL4 positive events) are readily distinguished from both cell debris and free bacteria released from disrupted vacuoles. Thus, phagosome preparations can be stained with specific antibodies followed by FITC-conjugated secondary antibodies and levels of phagosomal markers (FL1 histograms) can be easily determined by FACS.
(TIF)

Figure S5 Rac1 and p67^phox^ levels on Ndk-bead phagosomes. CellMask-labelled RAW cells were allowed to ingest BSA or Ndk coated 3 µm magnetic beads for 1 h. Bead containing phagosomes were then isolated by a magnet from crude preparations obtained as described in **Fig. S4**. Purified phagosomes were stained with Rac1 (**A**) or p67^phox^ (**B**) antibodies or irrelevant (control) antibody and FITC-conjugated secondary antibody. Samples were then washed and analyzed by FACS to quantify levels of FL1 signal on gated FL4 positive events, which correspond to true phagosomes. FL1 histograms showed decreased levels of Rac1 and p67^phox^ on Ndk-bead phagosomes relative to control BSA-bead.
(TIF)

Figure S6 Mtb Ndk inhibits ROS production in BMDM. **A**. Adherent cells on cover slips were stimulated with LPS then infected with Mtb strains expressing DsRed in presence of CM-DCFDA as described in **Fig. 5C**. Cells were then fixed and examined by confocal microscopy. Yellow signal (indicative of ROS production) is visible on phagosomes containing Mtb Ndk-AS but absent on those containing wild type and Ndk-S strains. **B**. Mean ± SD of positive phagosomes observed in 50–80 cells from three independent experiments.
(TIF)

Figure S7 Ndk has no apparent effect on the activation of p38 MAPK and ERK1/2. Adherent RAW cells were exposed to coated beads (MOI 5∶1) and incubated for 1 h at 37°C. Cells were then stimulated with 100 ng/mL LPS for 15 min to induce p38MAPK activation or 100 nM PMA for 30 min to induce ERK1/2 activation. Cell lysate were prepared in appropriate lysis buffer and subjected to SDS-PAGE and western blot analysis with anti-phospho-p38MAPK or anti-phospho-ERK1/2. Blots were then stripped and probed with antibodies to total p38MAPK or total ERK1/2.
(TIF)

Figure S8 Validation of gp91^phox^ inhibitor peptide. RAW macrophages were incubated in the presence of gp91^phox^ inhibitor peptide (gp91 INH) or its scrambled version (gp91 SCR) at a final concentration of 50 µM. Cells were infected 1 h later with Mtb Ndk-AS in the presence of 50 µM luminol and chemiluminescence was monitored as described in **Fig. 5**. The results obtained showed strong inhibition of ROS production by gp91 INH.
(TIF)

Figure S9 Ndk does not interfere with NO production in response to IFN- γ. RAW cells were exposed to coated beads (MOI 5∶1) and incubated for 1 h at 37°C. Cells were then left untreated or stimulated with IFN-γ for 24 h. NO production was then measured by the Griess reaction method.
(TIF)

Figure S10 Purity of recombinant Ndk and specificity of Ndk antibody. **A**. Expression and purification of rNdk was described in our previous work [17]. Different fractions of *E. coli* BL21 lysate expressing Ndk and two elution fractions following Ni-NTA affinity purification were resolved by 15% SDS-PAGE and total protein bands were visualized by Coomassie Blue stain. The elution fractions showed a single intense Ndk band at ∼15 kDa with no apparent contaminants. **B**. Purified Ndk fractions were pooled and dialyzed to remove imidazole. Aliquots of Ndk protein (100 ng and 200 ng) were resolved by SDS-PAGE along with lysate from RAW macrophages and then subjected to western blotting with rabbit anti-Ndk. The result shown demonstrates both the identity of purified Ndk and the specificity of the antibody since no band was detected in the lane loaded with RAW cell lysates.
(TIF)
