# Supplementary figures and images for: Mycobacterium tuberculosis Nucleoside Diphosphate Kinase Inactivates Small GTPases Leading to Evasion of Innate Immunity
Source: PLoS Pathog. 2013 Jul 18;9(7):e1003499. doi: 10.1371/journal.ppat.1003499 (PMC3715411; doi:10.1371/journal.ppat.1003499)

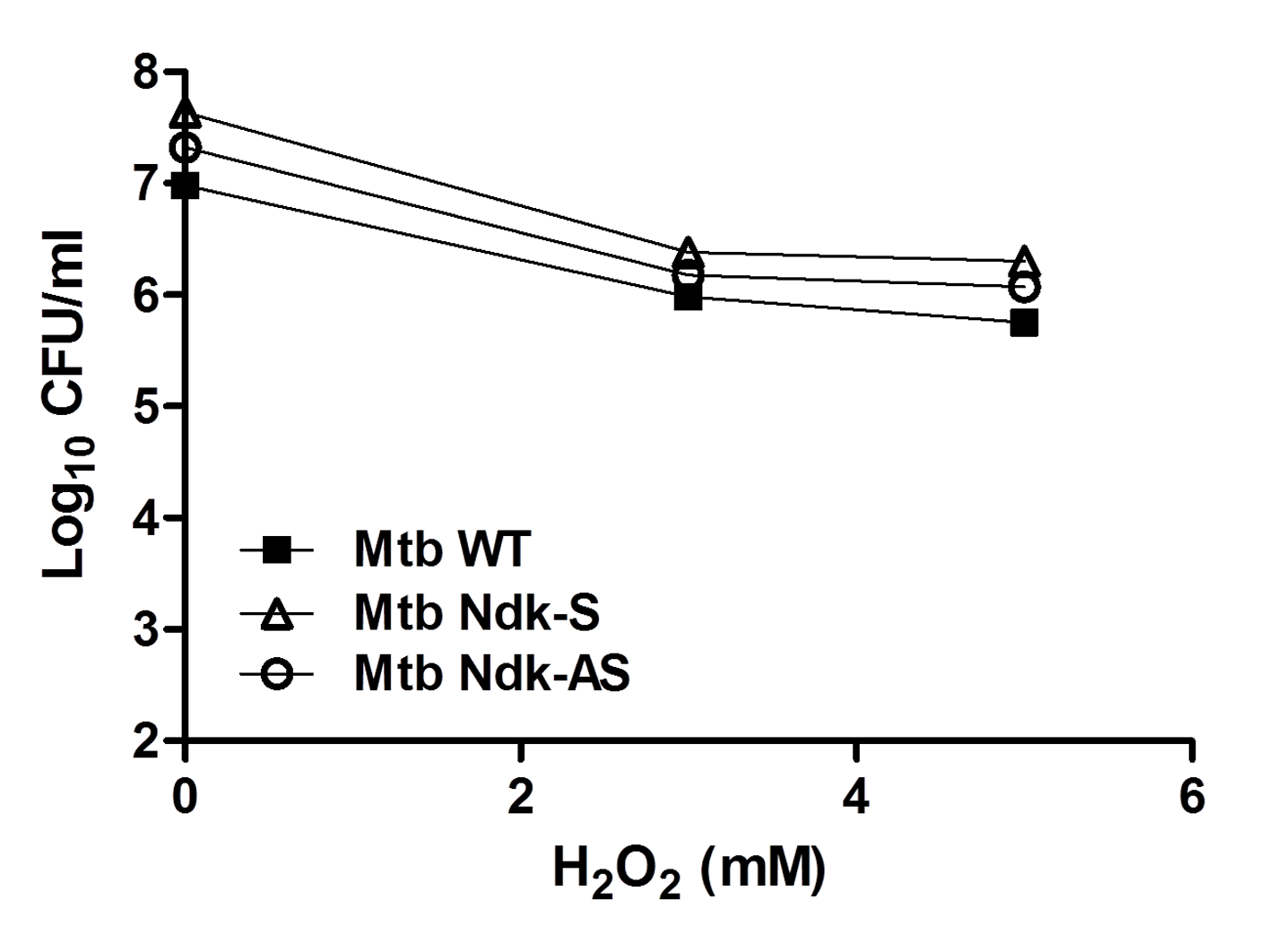

Supplement: Figure S1 — Equal number of exponentially growing bacteria (∼107/ml) were exposed to the indicated concentrations of H2O2 for 4 h. Thereafter 100 µl aliquots were plated in duplicates on 7H10 (WT Mtb) or 7H10 plus kanamycin (Mtb Ndk-S and -AS) for 3 weeks. Results are expressed as Log10 of CFUs. (TIF) [file ppat.1003499.s001.tif]

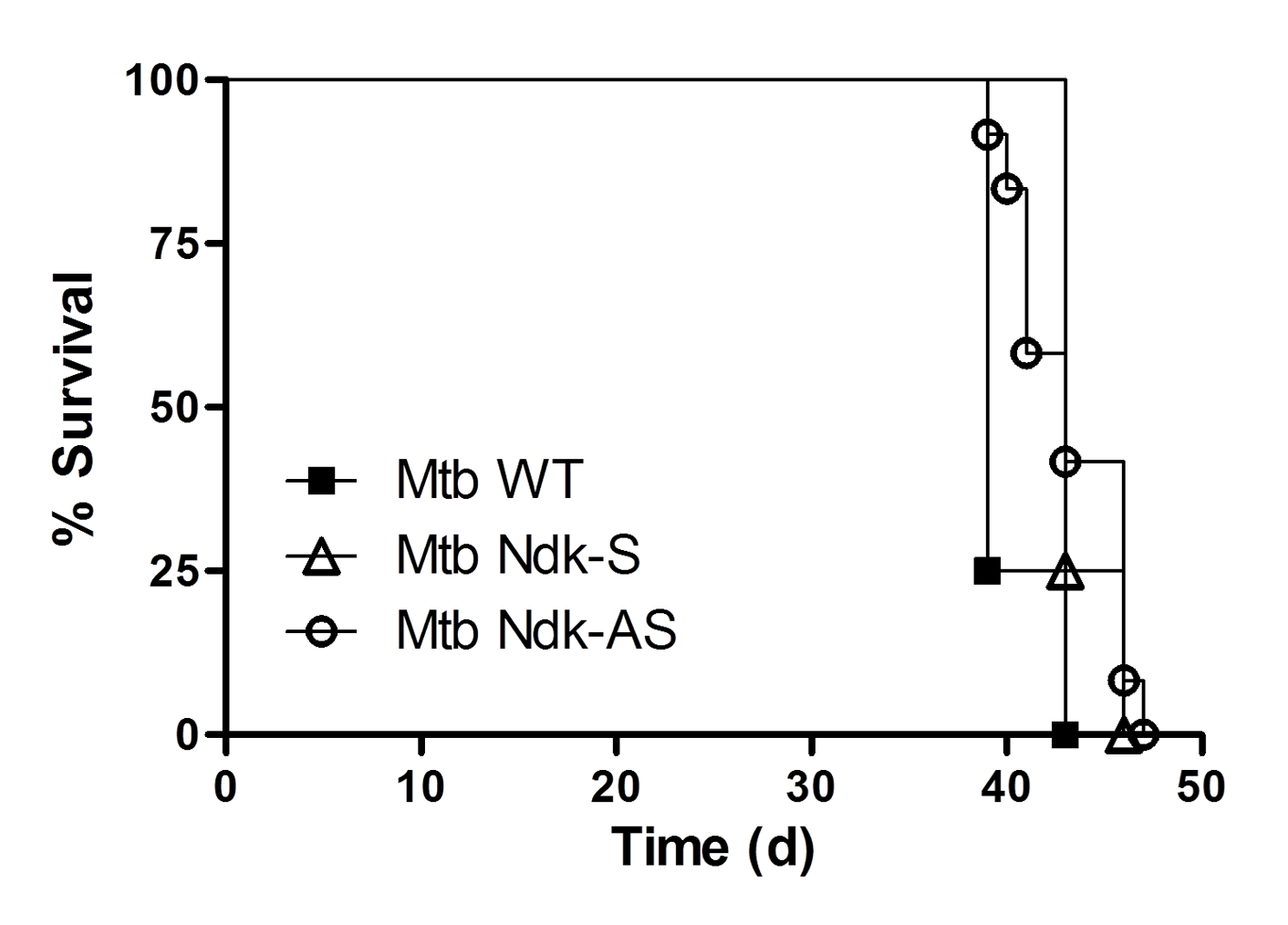

Supplement: Figure S2 — Fox Chase SCID mice (n = 10 per group) were infected with ∼150 bacteria by inhalation and survival was monitored over six weeks. (TIF) [file ppat.1003499.s002.tif]

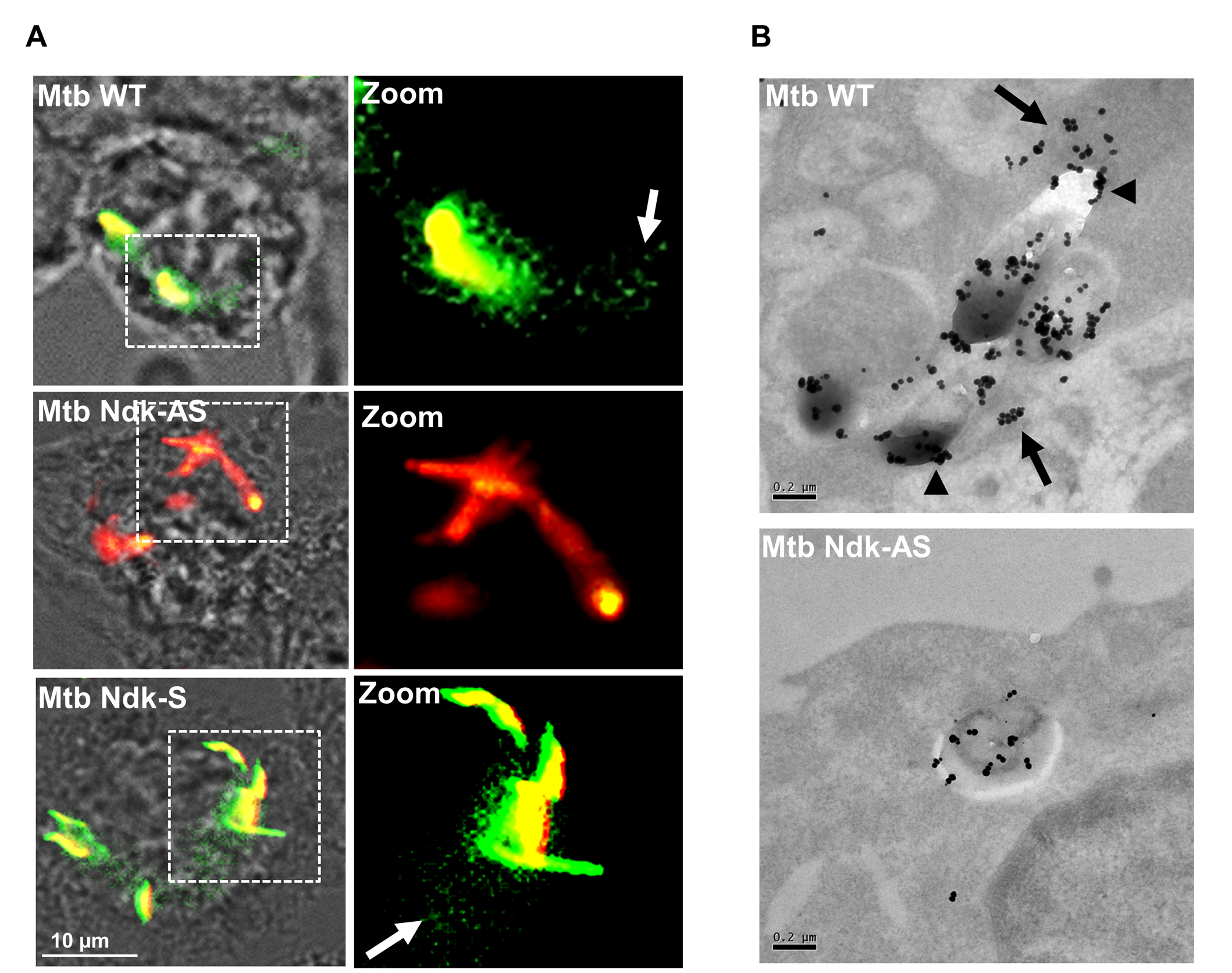

Supplement: Figure S3 — RAW 264.7 macrophages were infected with Mtb strains expressing DsRed (red fluorescent) at a MOI of 10∶1 and then washed thrice 2 h post infection to remove extracellular bacteria, then reincubated for additional 4 h. A) Cells were fixed/permeabilized, stained with rabbit Ndk antibodies and FITC-conjugated anti-rabbit IgG (green fluorescence), then examined by confocal microscopy. The images shown are the merge of green and red signals. The yellow signal reflects detection of Ndk (green) within the bacteria (red). Such signal is strong in cells infected with wild type and Ndk-S as both produce substantial levels of Ndk, but very weak in cells infected with Ndk-AS strain, which produces very little Ndk. White arrows indicate Ndk trafficking beyond bacterial phagosomes (green signal alone) in cells infected with wild type (WT) and Ndk-S strains. B) Macrophages infected with wild type Mtb or Mtb Ndk-AS were fixed with 4% paraformaldehyde, embedded in LR White resin then cut (60 nm sections) with a Leica EM UC6 microtome. Sections were collected on nickel grids and labelled with Ndk antibodies then F(ab′)2 of ultra-small goat-anti-rabbit IgG. Sections were then post-fixed in 2% glutaraldehyde and subjected to silver enhancement for gold labeling with Silver R-Gent SE-EM. Samples were then washed air dried and examined with a Tecnai 12 electron microscope. Arrowheads indicate Ndk localized on the phagosomal membrane and full arrows indicate translocated Ndk to the cytosol. Background signal was absent in control sections stained with secondary antibody alone. (TIF) [file ppat.1003499.s003.tif]

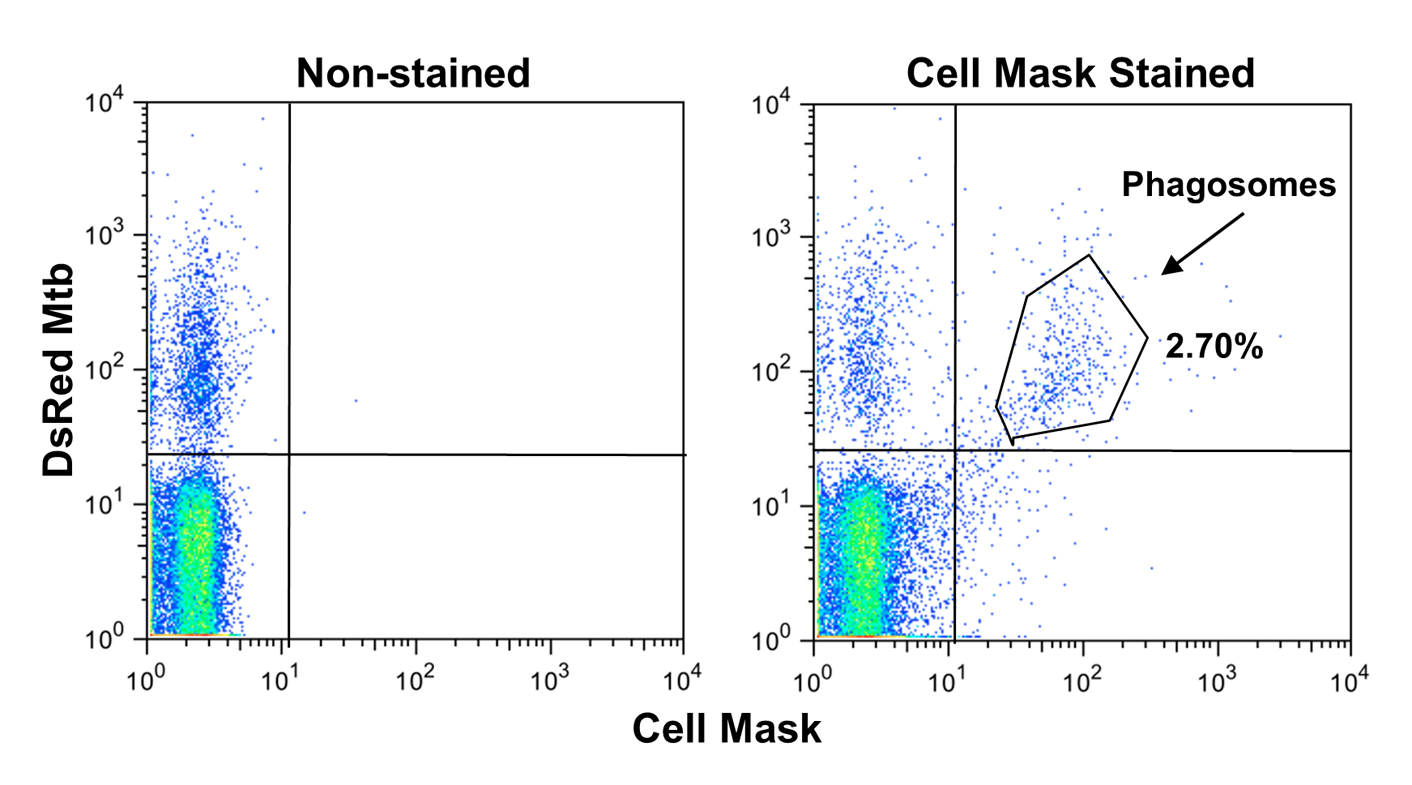

Supplement: Figure S4 — FACS analysis of Mtb phagosomes. Macrophage cell surface is labelled with CellMask Deep Red (detectable by FL4 channel) at 0.2 µg/ml for 5 min at 37°C prior to infection with DsRed mycobacteria (FL2). Then cells are treated with Trypsin-EDTA to remove non-ingested but partially attached bacteria. Thereafter, cells are homogenized in 20 mM HEPES buffer, pH 7.4 containing at, 0.25% sucrose, 0.1% BSA, and 0.5 mM EGTA. Homogenates were then centrifuged at 300× g for 2 min to remove nuclei and intact cells and the upper fractions were collected and centrifuged at 3,200× g for 10 min at 4°C. The pellets correspond to crude phagosome preparations where bacteria included in cell membrane-derived vacuoles (double FL2/FL4 positive events) are readily distinguished from both cell debris and free bacteria released from disrupted vacuoles. Thus, phagosome preparations can be stained with specific antibodies followed by FITC-conjugated secondary antibodies and levels of phagosomal markers (FL1 histograms) can be easily determined by FACS. (TIF) [file ppat.1003499.s004.tif]

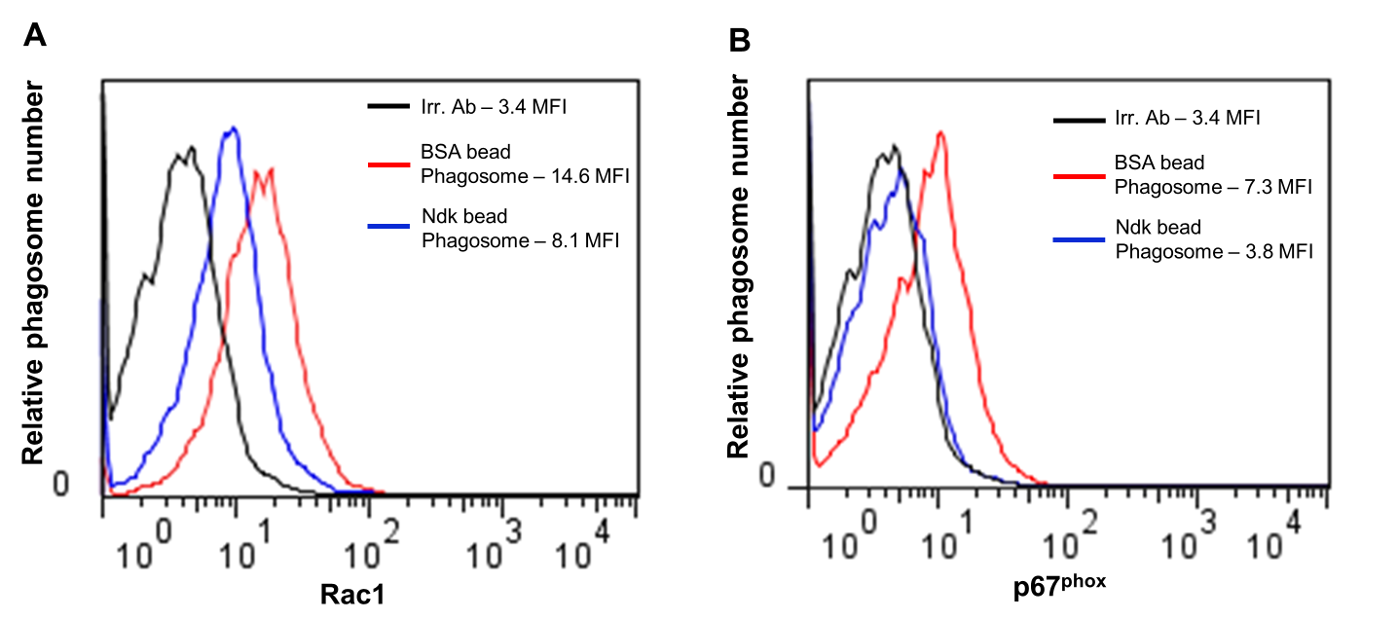

Supplement: Figure S5 — Rac1 and p67phox levels on Ndk-bead phagosomes. CellMask-labelled RAW cells were allowed to ingest BSA or Ndk coated 3 µm magnetic beads for 1 h. Bead containing phagosomes were then isolated by a magnet from crude preparations obtained as described in Fig. S4. Purified phagosomes were stained with Rac1 (A) or p67phox (B) antibodies or irrelevant (control) antibody and FITC-conjugated secondary antibody. Samples were then washed and analyzed by FACS to quantify levels of FL1 signal on gated FL4 positive events, which correspond to true phagosomes. FL1 histograms showed decreased levels of Rac1 and p67phox on Ndk-bead phagosomes relative to control BSA-bead. (TIF) [file ppat.1003499.s005.tif]

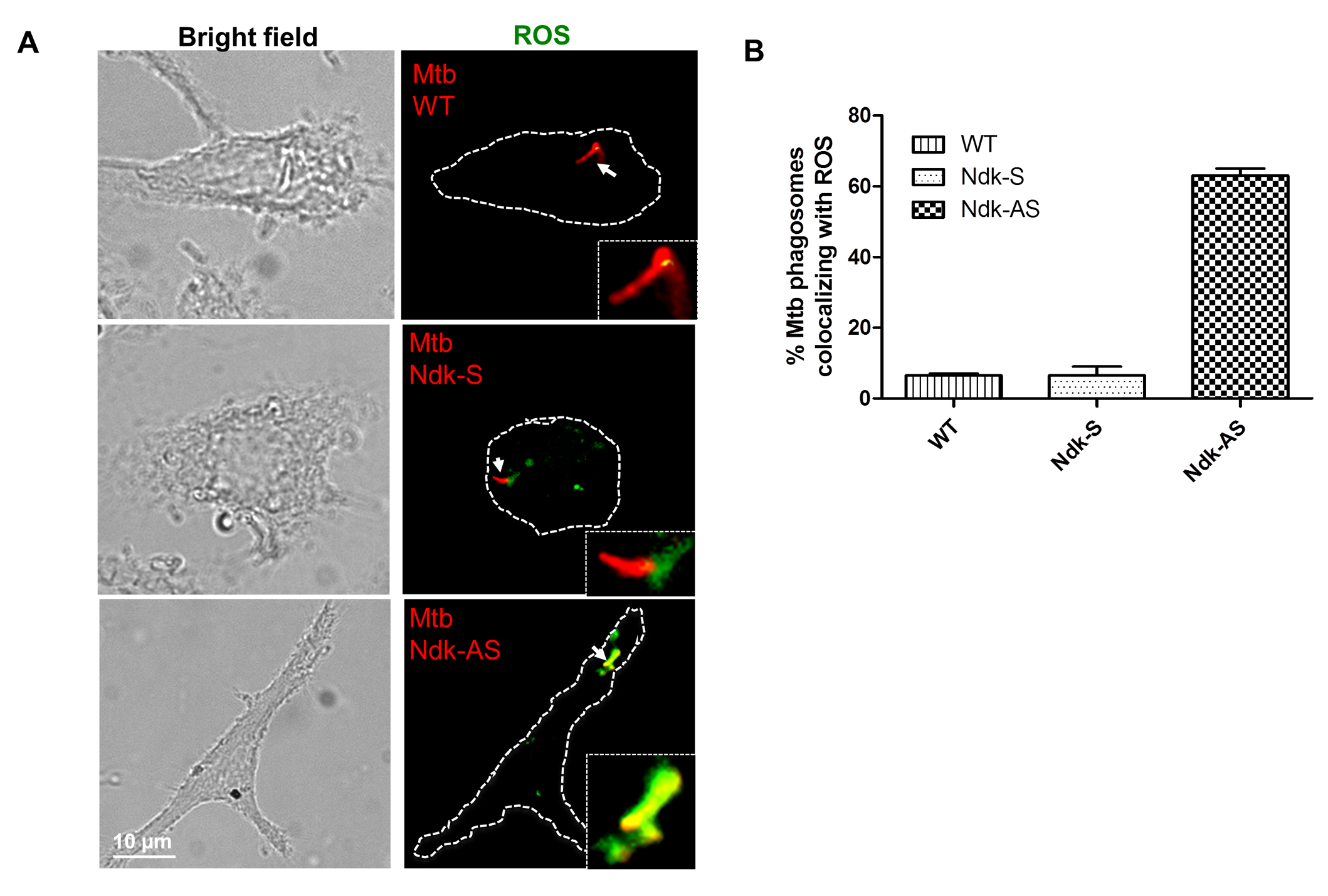

Supplement: Figure S6 — Mtb Ndk inhibits ROS production in BMDM. A. Adherent cells on cover slips were stimulated with LPS then infected with Mtb strains expressing DsRed in presence of CM-DCFDA as described in Fig. 5C . Cells were then fixed and examined by confocal microscopy. Yellow signal (indicative of ROS production) is visible on phagosomes containing Mtb Ndk-AS but absent on those containing wild type and Ndk-S strains. B. Mean ± SD of positive phagosomes observed in 50–80 cells from three independent experiments. (TIF) [file ppat.1003499.s006.tif]

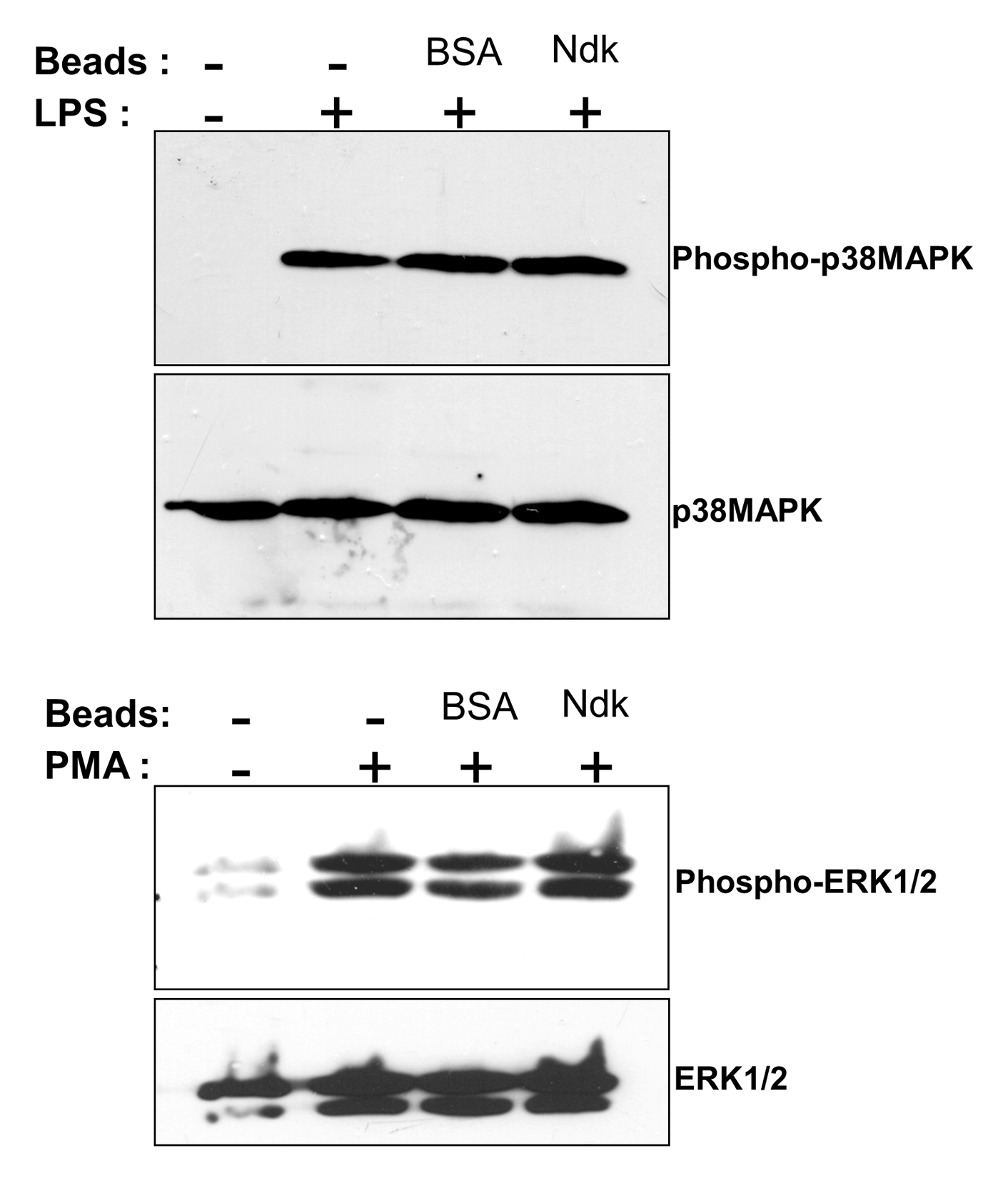

Supplement: Figure S7 — Ndk has no apparent effect on the activation of p38 MAPK and ERK1/2. Adherent RAW cells were exposed to coated beads (MOI 5∶1) and incubated for 1 h at 37°C. Cells were then stimulated with 100 ng/mL LPS for 15 min to induce p38MAPK activation or 100 nM PMA for 30 min to induce ERK1/2 activation. Cell lysate were prepared in appropriate lysis buffer and subjected to SDS-PAGE and western blot analysis with anti-phospho-p38MAPK or anti-phospho-ERK1/2. Blots were then stripped and probed with antibodies to total p38MAPK or total ERK1/2. (TIF) [file ppat.1003499.s007.tif]

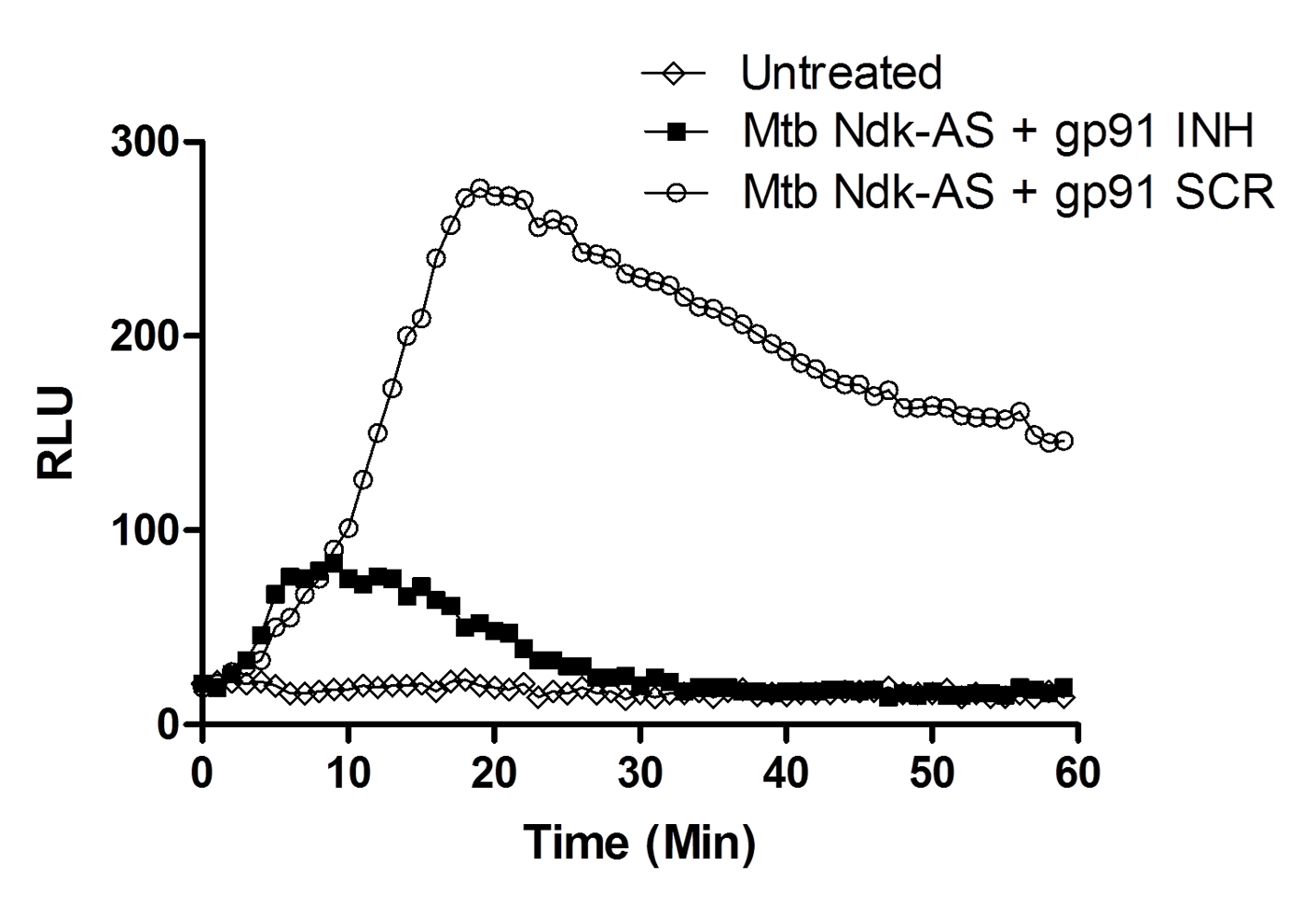

Supplement: Figure S8 — Validation of gp91phox inhibitor peptide. RAW macrophages were incubated in the presence of gp91phox inhibitor peptide (gp91 INH) or its scrambled version (gp91 SCR) at a final concentration of 50 µM. Cells were infected 1 h later with Mtb Ndk-AS in the presence of 50 µM luminol and chemiluminescence was monitored as described in Fig. 5 . The results obtained showed strong inhibition of ROS production by gp91 INH. (TIF) [file ppat.1003499.s008.tif]

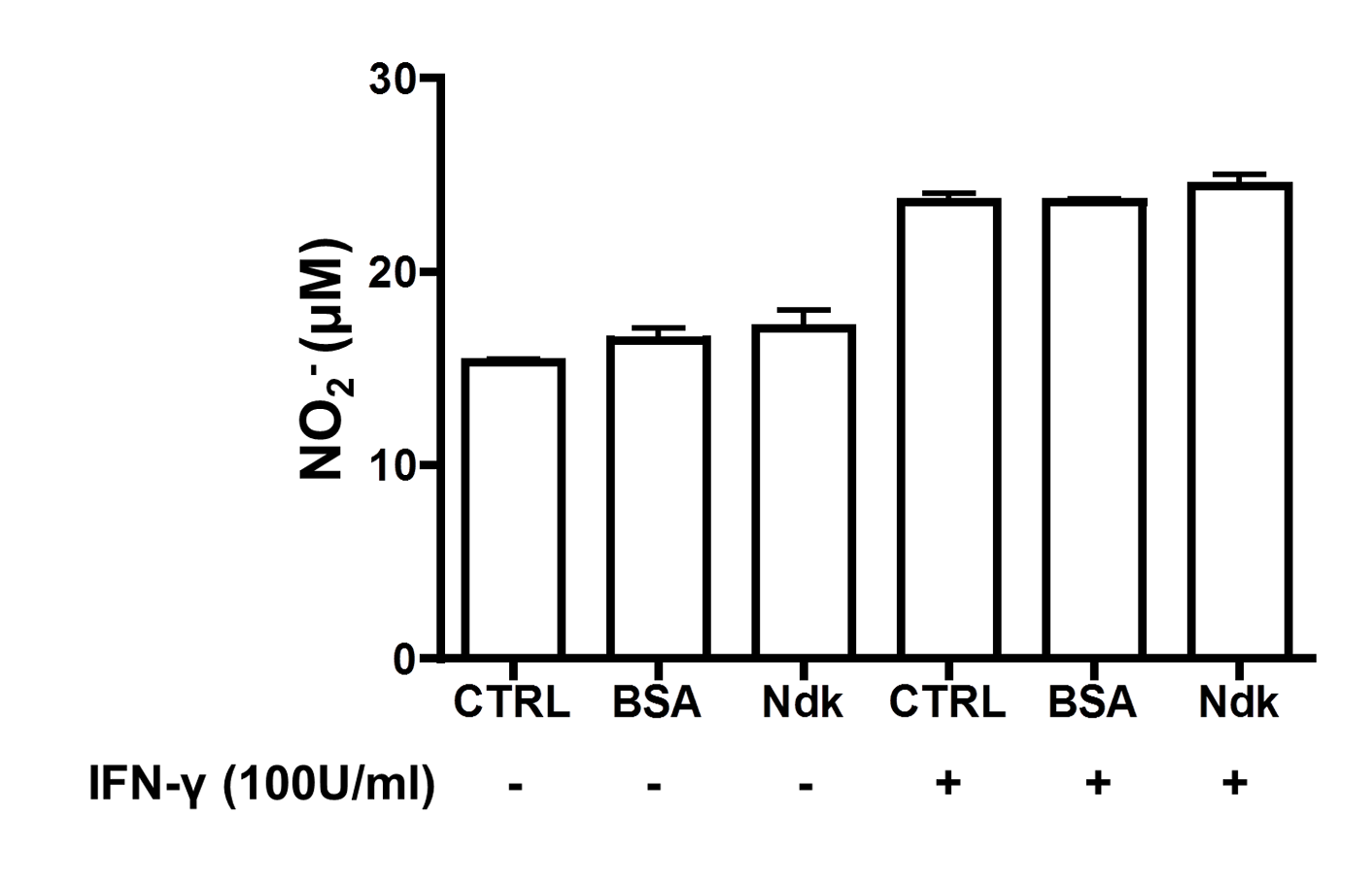

Supplement: Figure S9 — Ndk does not interfere with NO production in response to IFN- γ. RAW cells were exposed to coated beads (MOI 5∶1) and incubated for 1 h at 37°C. Cells were then left untreated or stimulated with IFN-γ for 24 h. NO production was then measured by the Griess reaction method. (TIF) [file ppat.1003499.s009.tif]

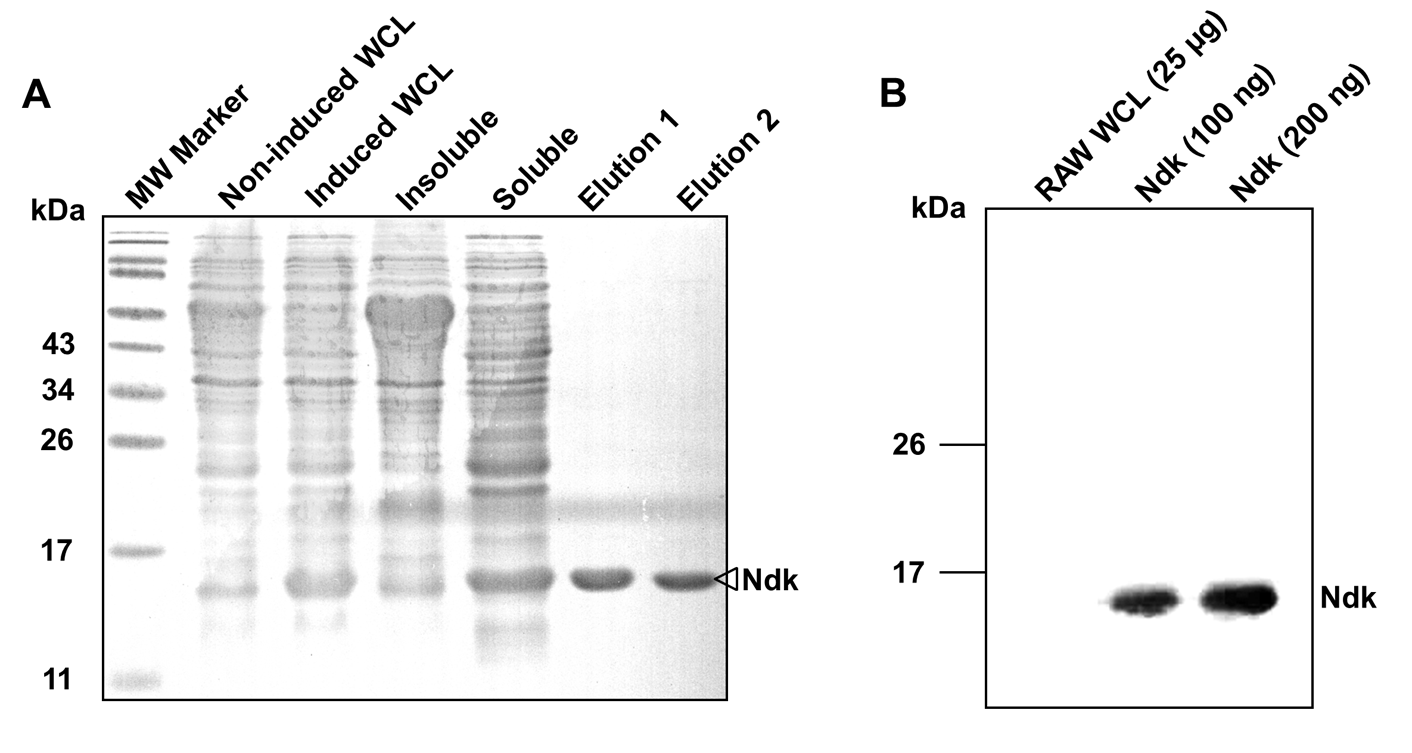

Supplement: Figure S10 — Purity of recombinant Ndk and specificity of Ndk antibody. A. Expression and purification of rNdk was described in our previous work [17]. Different fractions of E. coli BL21 lysate expressing Ndk and two elution fractions following Ni-NTA affinity purification were resolved by 15% SDS-PAGE and total protein bands were visualized by Coomassie Blue stain. The elution fractions showed a single intense Ndk band at ∼15 kDa with no apparent contaminants. B. Purified Ndk fractions were pooled and dialyzed to remove imidazole. Aliquots of Ndk protein (100 ng and 200 ng) were resolved by SDS-PAGE along with lysate from RAW macrophages and then subjected to western blotting with rabbit anti-Ndk. The result shown demonstrates both the identity of purified Ndk and the specificity of the antibody since no band was detected in the lane loaded with RAW cell lysates. (TIF) [file ppat.1003499.s010.tif]
